# Keratinocyte-derived VEGF-A is an essential pro-migratory autocrine mediator, acting through the KDR/GEF-H1/RhoA pathway

**DOI:** 10.3389/fcell.2025.1601887

**Published:** 2025-07-17

**Authors:** Vida Maksimoska, Qinghong Dan, Neetu Rambharack, Katalin Szászi

**Affiliations:** ^1^ Keenan Research Centre for Biomedical Science of the St. Michael’s Hospital, Unity Health Toronto, Toronto, ON, Canada; ^2^ Institute of Medical Sciences, University of Toronto, Toronto, ON, Canada; ^3^ Laboratory Medicine and Pathobiology, University of Toronto, Toronto, ON, Canada; ^4^ Department of Surgery, University of Toronto, Toronto, ON, Canada

**Keywords:** Rho GTPase, keratinocytes, skin biology, cell migration, RhoGEF, tumor necrosis Factor α

## Abstract

**Introduction:**

Keratinocytes proliferate, migrate and differentiate to achieve skin re-epithelialization following injury. They also secrete soluble mediators to induce inflammation and orchestrate restoration of the skin barrier. However, dysregulated mediator release can cause sustained inflammation, leading to pathological healing. The small GTPase RhoA is key for cell migration, but the molecular mechanisms controlling Rho proteins in keratinocytes remain incompletely characterized. The overall objective of the current study was to explore the connection between inflammation-induced keratinocyte mediator release and enhanced migration, and to identify specific RhoA regulators involved.

**Methods:**

The study was done using HaCat cells and primary adult keratinocytes. A multiplex cytokine panel was used to simultaneously detect 48 mediators secreted from TNFα-stimulated HaCat cells. Cell migration was followed using live timelapse imaging. Target proteins were silenced using siRNA or inhibited with drugs. RhoA and GEF-H1 activation were detected using affinity precipitation assays with GST-RBD or GST-RhoA (G17A). Key proteins were visualized using immunohistochemistry in an MC903-induced mouse model of atopic dermatitis.

**Results:**

We showed that keratinocytes secreted an array of soluble factors, including VEGF-165. Secretion of VEGF-165 was augmented by TNFα through SP1, HIF1α and NFκB. TNFα or VEGF-165 potently augmented HaCaT collective migration. Depletion of VEGF-A or VEGF Receptor2 (referred to as Kinase Insert Domain Receptor, KDR) or inhibition of RhoA reduced basal migration and prevented the pro-migratory effect of TNFα. Both VEGF-165 and TNFα increased KDR phosphorylation. VEGF-165 activated GEF-H1 (ArhGEF2) through KDR and ERK1/2. VEGF-165 also promoted GEF-H1 phosphorylation on S886. GEF-H1 depletion reduced VEGF-induced RhoA activation, slowed migration, and inhibited TNFα-induced VEGF-165 release. Finally, the epidermis in a mouse atopic dermatitis model had increased active RhoA, phospho-GEF-H1 and phospho-KRD levels.

**Discussion:**

We showed that VEGF-A is a crucial paracrine factor, essential for basal and TNFα-induced keratinocyte migration. VEGF-165 activated RhoA through KDR and GEF-H1, and this pathway was upregulated in skin inflammation. Thus, GEF-H1 is critical for keratinocyte migration and VEGF-A secretion. Targeting the KDR/GEF-H1/RhoA pathway may reduce keratinocyte inflammatory responses, providing benefits in inflammatory skin disease.

## 1 Introduction

The outermost layer of the skin, the epidermis is a stratified squamous epithelium predominantly composed of keratinocytes organized in 4 layers: stratum basale, spinale, granulosum and corneum. The epidermis is the first line of defense against harmful chemicals, mechanical stimuli, ultraviolent light and pathogens (Yousef et al., 2024), and is crucial for immune responses, inflammation and wound healing. The epidermis undergoes continuous renewal (Fukuda et al., 2025). Basal keratinocytes proliferate and then move outward into the suprabasal layers and undergo a terminal differentiation process. They gradually lose their proliferative capacity, modify their cell-cell adhesions and keratin expression profile and produce cornified envelope proteins. Terminally differentiated keratinocytes (corneocytes) lose their nuclei and are eventually shed. Although numerous studies provided new insights into mechanisms of skin regeneration and wound healing, many details of these complex events remain unknown. Efficient re-epithelialization following injury requires the upregulation of proliferation, migration and differentiation of keratinocytes (Pastar et al., 2014). Indeed, during wound healing, basal and suprabasal keratinocytes transition to a migratory and hyperproliferative phenotype promoted by inflammatory mediators and growth factors (reviewed in (Seeger and Paller, 2015; Piipponen et al., 2020)). Keratinocytes are a major source of antimicrobial agents, chemokines, cytokines and growth factors, which coordinate efficient healing via crosstalk with immune cells, fibroblasts and the endothelium (Piipponen et al., 2020; Simmons and Gallo, 2024) through paracrine effects. Keratinocyte-derived secreted signals can also affect the keratinocytes through autocrine effects, i.e., when the same cell responds to mediators it releases. These autocrine effects, however, remain less explored. One important inflammatory cytokine produced by both keratinocytes and immune cells is Tumor Necrosis Factor (TNF)α (Wakefield et al., 1991). Keratinocytes are both sources and targets of TNFα (Köck et al., 1990). This pleiotropic cytokine is essential for skin homeostasis and its synthesis and release are rapidly induced by injury (Kristensen et al., 1993; Bashir et al., 2009a; Aufiero et al., 2007; Kondo and Ohshima, 1996). In the normal skin, TNFα is found predominantly at the basal layer, and its expression is increased by harmful environmental insults, such as ultraviolet B (UVB) light and pathogens (Köck et al., 1990; Aufiero et al., 2007; Bashir et al., 2009b) and pathogens and during wound healing (Kondo and Ohshima, 1996; Ettehadi et al., 1994). Importantly, while TNFα has many pro-healing effects, its sustained elevation contributes to disease. Accordingly, TNFα was found to be elevated in various skin diseases, such as psoriasis and allergic contact dermatitis (Ettehadi et al., 1994; Kaplan et al., 2012). Global gene expression profiling in TNF-stimulated keratinocytes revealed increased transcription of a multitude of inflammatory and pro-healing genes, including cytokines and chemokines and growth factors (Banno et al., 2004). TNFα was also shown to promote keratinocyte migration in part via the upregulation of actin cytoskeleton regulators and integrins (Banno et al., 2004).

Rho family small GTPases are central regulators of the actin cytoskeleton. They play crucial roles in keratinocyte biology, as they affect differentiation, cell adhesion and migration (McMullan et al., 2003; Grossi et al., 2005). During migration, dynamic reorganization of the cytoskeleton is controlled by an interplay between activated Rho family small GTPases (Raftopoulou and Hall, 2004; Machacek et al., 2009), leading to front-back polarization, formation of actin-containing structures such as leading-edge protrusions and changes in cell-substrate adhesion, vesicle trafficking and transcription. Although lamellipodium extension at the leading edge was initially mostly associated with Rac activation, and RhoA was suggested to control acto-myosin contractility in the cell body and at the rear (Raftopoulou and Hall, 2004; Machacek et al., 2009), live imaging with biosensors revealed a more complex situation by demonstrating that RhoA is also active at the front protrusions (Pertz et al., 2006). Epithelial cells, including keratinocytes, migrate collectively, maintaining cell-cell adhesions, which also requires well-coordinated Rho GTPase activation and inactivation (Zegers and Friedl, 2014). Indeed, primary keratinocytes from mice with keratinocyte-specific RhoA deletion exhibited slower migration and reduced directional persistence (Jackson et al., 2011).

Despite the importance of Rho proteins in keratinocyte biology, only few studies explored their upstream regulators. Rho proteins cycle between GTP-bound (active) and GDP-bound (inactive) forms, regulated by guanine nucleotide exchange factors (GEFs), and GTPase-activating proteins (GAPs) (Cherfils and Zeghouf, 2013; Hodge and Ridley, 2016). GEFs promote exchange of GDP to GTP, leading to Rho activation (Goicoechea et al., 2014). Over 80 RhoGEFs and 70 RhoGAPs have been identified and many questions of their roles remain unanswered. In keratinocytes, GEF-H1 (ARHGEF2) was recently shown to bind to the junction associated scaffold Plakophilin 4, and control cortical acto-myosin (Müller et al., 2024). Further, knockdown of a specific Rho GAP, ARHGAP29 in keratinocytes caused significant delay in scratch wound closure, indicating a crucial need for a fine balance between activating and inactivating input (Rhea et al., 2024).

The overall objective of the current study was to explore the connection between inflammation-induced mediator release and migration in keratinocytes, and to establish how key mediators promote migration-related RhoA activation. We show that TNFα induces the release of VEGF-165, that is essential for efficient basal and TNFα-stimulated migration. VEGF-165 acts through KDR and GEF-H1/RhoA signalling. Interestingly, VEGF-165 release itself is GEF-H1-dependent, suggesting a self-augmenting pro-migratory loop.

## 2 Materials and methods

### 2.1 Materials and antibodies

Human Recombinant VEGF-A-165 (VEGF-165) was obtained from two sources: Cell Signaling Technology (Danvers, MA) (Cat#48143S) and MedChemExpress (Monmouth Junction, NJ) (Cat#HY-P7110A). TNFα was from MedChemExpress (Cat#HY-P7058). Chemical inhibitors were purchased from the following sources: Echinomycin (Cat#5520), Rhosin Hydrochloride (Cat#5003) and Actinomycin D (Cat # 1229) were from Tocris Bioscience/Biotechne (Minneapolis, MN); Mithramycin A (Cat#11434) and Bay11-7085 (Cat#14795) from Cayman Chemicals (Ann Arbor, MI); SU5408 (Cat#103002) from MedChemExpress; PD184352 (Cat #S1020) from Selleck Chemicals (Houston, TX). Bovine serum albumin (BSA) was from BioShop Canada (Burlington, On). The Complete Mini Protease inhibitor and PhosSTOP Phosphatase Inhibitor tablets were from Roche Diagnostics (Laval, QC).

Proteins were detected using the following antibodies: GEF-H1 (Cat# 4076, RRID:AB_2060032), phospho-S886 (pS886)-GEF-H1 (Cat#14143, RRID:AB_2798402), RhoA (Cat# 2117, RRID:AB_10693922), VEGF-A (Cat#65373); pERK1/2 (Cat# 9102, RRID:AB_330744), phospho-pERK1/2 (Cat#4370, RRID:AB_2315112), Phospho-Myosin Light Chain 2 (Thr18/Ser19) (Cat# 95777, RRID:AB_3677547) from Cell Signalling Technologies (Danvers, MA); phospho-VEGF Receptor 2 (Tyr951) (referred to as phospho-KDR) (Cat# PA5-104882, RRID:AB_2816355), VEGF Receptor 1 (Cat #64094, RRID:AB_3697182) and Glyceraldehyde-3-phosphate dehydrogenase (GAPDH) (Cat# 39-8600, RRID:AB_2533438) from ThermoFisher Scientific (Whitby, ON); IKBα (Cat# 10268-1-AP, RRID:AB_2151423) and VEGFR2/KDR (Cat# 26415-1-AP, RRID:AB_2756527) from Proteintech (Rosemont, IL). The active RhoA-GTP (Cat# 26904, RRID:AB_1961799) antibody was from NewEast BioSciences (King of Prussia, PA). We validated this antibody using immunofluorescent staining of cells treated with C3 toxin, that inhibits RhoA by ADP-ribosylation (Cytoskeleton Cat# CT03-A) (negative control) or a RhoA Activator II (Cytoskeleton Cat# CN03), that stabilizes RhoA in a GTP-bound state leading to strong staining (positive control) (not shown). The HRP-linked anti-rabbit IgG Cat# 7074, RRID:AB_2099233) and anti-mouse IgG (Cat# 7076, RRID:AB_330924) and Alexa Fluor 555-labelled anti-rabbit IgG (Cat# 4413, RRID: AB_10694110) were from Cell Signaling Technology. 4, 6-diamidino-2-phenylindole (Dapi) Cat#10236276001 was from Millipore Sigma (Burlington, ON).

### 2.2 Cell culture

HaCaT cells, a spontaneously transformed human keratinocyte cell line (male) was obtained from Antibody Research Corporation (St. Peters, MO, United States) (Product Code 116027, RRID:CVCL_0038). HaCaT cells were cultured in DMEM medium containing 4500 mg/L D-glucose, 1.8 mM CaCl_2_, pyridoxine HCl, and L-glutamine but lacking sodium pyruvate, and supplemented with 10% fetal bovine serum and 1% penicillin-streptomycin. Adult Primary Normal Human Epidermal Keratinocytes (HEKa) (female) were from ThermoFisher Scientific (cat #: C0055C). HEKa cells were cultured in EpiLife™ Medium, containing 60 μM calcium (cat# MEPI500CA), supplemented with EpiLife™ Defined Growth Supplement (cat#S0125). All cell culture media and reagents were purchased from ThermoFisher Scientific.

### 2.3 MTT cell viability assay

An MTT Assay Kit (Cat# ab211091 from Abcam (Toronto, ON) was used following the manufacturer’s instructions. HaCaT cells were grown in 24-well plates overnight. The medium was then changed to an antibiotic-free DMEM containing 20 ng/mL TNFα or one of the inhibitors, or TNFα and the indicated inhibitors: Echinomycin (HIF1α inhibitor, 20 µM), Mithramycin A (Sp1 inhibitor, 10 µM) or Bay11-7058 (NFκB inhibitor, 10 µM) for 16 h. At the end of the treatment, the medium was replaced with a 1:1 mixture of DMEM and MTT reagent for 3 h at 37°C. Following incubation, the MTT/DMEM mixture was removed, and 0.5 mL of MTT solvent was added to each well, and the plates were shaken at room temperature for 15 min. Absorbance was measured at 590 nm using a SpectraMax plate reader. Data were collected in triplicates (n = 3). The background (cell culture medium only) from each sample was subtracted and the percentage of cell viability was calculated by expressing the values for the treated cells as the percentage of the untreated control (set to 100%).

### 2.4 Protein silencing

Short-interfering RNA (siRNA) oligonucleotides were purchased from the following sources: Non targeting control (non-related, NR) (Cat #D-001810-01) and GEF-H1 (Cat #J-009883-06) were from Horizon Discovery (Lafayette, LA); VEGF-A (Cat # ZO2689) from GenScript (Piscataway, NJ), and VEGFR2/KDR (Cat# SR320782) from OriGene Technologies (Rockville, MD). Cells were transfected with 100 nM siRNA using Lipofectamine™ RNAiMAX (Invitrogen). Downregulation was verified using Western blotting.

### 2.5 Cell migration assay

To follow migration, HaCaT cells were grown to confluence on a glass plate (MatTek, Ashland, MA) within a removable two-well culture insert (Cat# 81176) (Ibidi, Gräfelfing, Germany). For migration experiments with protein knockdown, transfection was done in 6-well plates, and 24 h later cells were trypsinized and plated in Ibidi chambers on the MatTek glass plates for an additional 24 h. At confluence, the Ibidi chamber was removed, generating a gap into which the cells migrated. Treatments were applied at the time of the removal of the chamber. Cells were imaged using a VivaView FL Incubator Microscope (Olympus, Hachioji, Tokyo) and the Metamorph software, allowing visualization of multiple plates and areas at each time point. Time-lapse imaging was performed for 24 h, with images captured every 20 min. Gap closure was analyzed using sequential images and the T-scratch software (CSE Lab, ETH Zürich). Images were analyzed by calculating the percentage of the area covered by cells over time (Gebäck et al., 2009).

### 2.6 Cytokine array

**FIGURE 1:**
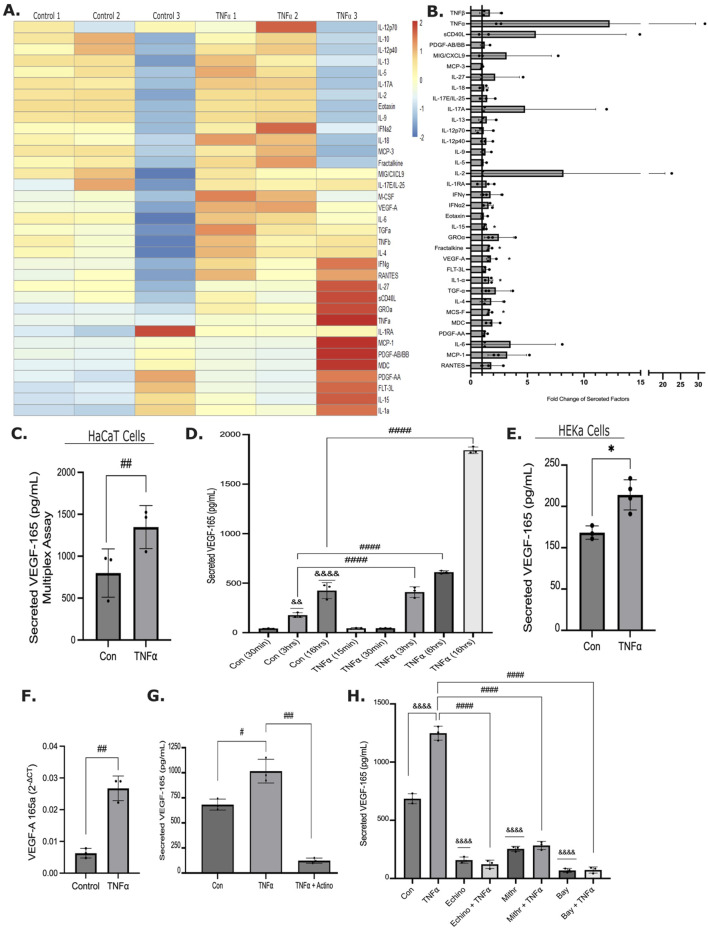
TNFα stimulates the release of soluble factors, including VEGF-165. **(A)** HaCat cells were treated with 20 ng/mL TNFα for 16 h, and the secretome was analyzed using a Human Cytokine/Chemokine 48-Plex Array. The heatmap shows z-scores for each sample. Corresponding numbers indicate control-TNFα pairs from the same experiment. **(B)** Fold change in the indicated soluble factors from the multiplex array was calculated taking the average of the controls as 1. *p < 0.05 (one sample t-test, n = 3) **(C)** VEGF-165 concentration changes in the HaCat cell supernatant measured by the multiplex array. **(D)** HaCat cells were treated with or without TNFα for the indicated times, and VEGF-165 was measured in the conditioned media using ELISA (n = 3, one-way Anova: ####p < 0.0001 vs. the indicated conditions; one-sample t-test: &&p < 0.01 vs. 1 and &&&& p < 0.0001 vs. 1). **(E)** HEKa cells were treated with TNFα for 16 h, and VEGF-165 in the supernatant was measured as in D (n = 3, paired t-test: ##p < 0.01). **(F)** HaCaT cells were treated with TNFα for 24 h, and the mRNA level of VEGF-A-165 (isoform a) was determined by PCR using GAPDH as the reference standard. (n = 3 t-test: ##p < 0.01). **(G)** HaCaT cells were treated with TNFα, with or without Actinomycin D for 16 h, and VEGF-165 was measured in the conditioned media as in C (n = 3, one-way ANOVA: #p < 0.05, ###p < 0.001). **(H)** HaCaT cells were treated for 16 h with TNFα alone, with the inhibitors alone, or with inhibitor + TNFα, as indicated. Inhibitors used: Echinomycin (20 µM), Mithramycin A (10 µM), Bay11-7058 (10 µM). VEGF-165 levels in the conditioned media were determined as in C (n = 3, one way ANOVA: &&&& p < 0.0001vs. control (30 min), ####p < 0.0001 vs. the indicated condition).

HaCaT cells were grown to confluence in a 12-well plate, then the medium was changed to a serum/antibiotic free medium and cells were treated with 20 ng/mL TNFα for 16 h. For experiments with protein silencing, transfection was done for 24 h prior to treatment with TNFα (16 h). Samples from the medium in each well were collected and centrifuged at 1,000x g for 2 min, and the conditioned media was stored a −20°C. The samples were shipped on dry ice to Eve Technologies (Calgary, AB, Canada) for measurement using a Human Cytokine/Chemokine 48-Plex Discovery Assay Array. Cytokines below the detection level were disregarded. For analyzing the data, the background (values measured in the medium alone) was subtracted from the raw values for each cytokine. In Figure 1A fold changes were used to calculate z-scores (indicating the number of standard deviations a data point is from the mean). For evaluating the effect of GEF-H1 silencing in Figure 7, data for each repeat in the treatment groups were averaged, then fold changes were calculated and used for calculating and plotting Z scores. The heatmap with the Z-scores was generated using R Studio Statistical software (version 4.4.2, R core team 2023, and Heatmap R package version 1.0.12, “pheatmap” plugin) (Kolde, 2018).

### 2.7 Quantification of VEGF-165

The Quantikine Human VEGF-165 Immunoassay kit was used according to the manufacturer’s instructions (R&D systems-Biotechne, Cat #:DVE00). Briefly, HaCaT or HEKa cells were grown in a 6-well or 12-well plate, respectively, to 80% confluence. For HaCaT cells, the regular culture medium was changed to DMEM containing 1% fetal bovine serum to ensure stability of VEGF. For HEKa cells, the medium was changed to supplement free EpiLife™ Medium. Where indicated, cells were treated with TNFα or inhibitors alone, or with TNFα and one of the inhibitors as follows: Echinomycin (20 µM), Mithramycin A (10 µM) or Bay11-7058 (10 µM). Conditioned media from each well was collected and centrifuged at 1,000x g for 2 min and stored at −20°C. For time course experiments, samples from the conditioned media were collected at the indicated times and stored at −20. For assessment of VEGF-165 in migrating cells, the cells were grown in a 6 well plate in three large Ibidi chambers (Cat #80466) per well, then at confluence the chambers were removed, and culture media with or without TNFα was added. The conditioned media was collected from migrating cells at 15 min, 8 h and 16 h. Samples were analyzed as above. The ELISA assay was conducted for all samples at the same time and measured using a Spectramax340 microplate reader at 450 nm wavelength. The corresponding concentration of VEGF-165 was determined using a standard curve and was expressed as pg/mL.

### 2.8 Western blotting

This was done as in our previous publications (Dan et al., 2019; Venugopal et al., 2024). Briefly, following the indicated treatments, HaCaT cells were lysed using ice cold lysis buffer (100 mM NaCl, 30 mM HEPES pH 7.5, 20 mM NaF, 1 mM EGTA, 1% Triton X-100, 1 mM Na3VO4, 1 mM PMSF, supplemented with Complete Mini Protease inhibitor and PhosSTOP Phosphatase Inhibitor). Lysates were centrifuged and protein concentration was determined using the BCA assay (Thermo Fisher/Pierce Biotechnology). Equal amount of protein of each sample were separated using SDS-PAGE and transferred to nitrocellulose. Blots were blocked in 5% bovine serum albumin and incubated with the primary antibody overnight at 4°C at 1:1,000. Following extensive washing, blots were incubated with the corresponding peroxidase-conjugated secondary antibodies. Signal was visualized using the enhanced chemiluminescence (ECL) method (BioRad, Hercules, CA). ECL signal was captured using a BioRad ChemiDoc Imaging system and densitometry was performed using ImageLab (Version 6.1.0 Build 7.2020). GAPDH was used as loading control. Blots were either stripped and redeveloped or were cut following transfer, and the corresponding parts were simultaneously developed with specific antibodies. Full blots for each figure are shown in the supplemental figure.

### 2.9 RhoA activation assay

RhoA activation was followed using affinity precipitation with GST-RBD (RhoA-binding domain: amino acids 7-89 of Rhotekin), as in our earlier studies (Venugopal et al., 2024). The preparation of the GST-RBD beads has been described in Waheed et al. (2012) Confluent HaCaT cells were treated as indicated, then lysed with ice-cold assay buffer containing 100 mM NaCl, 50 mM Tris base (pH 7.6), 20 mM NaF, 10 mM MgCl_2_, 1% Triton X-100, 0.5% deoxycholic acid, 0.1% SDS, 1 mM Na_3_VO_4_ and protease inhibitors. The samples were centrifuged and aliquots for determining total RhoA were reserved. The remaining supernatants were incubated with GST-RBD at 4°C for 45 min, followed by extensive washing. Aliquots of total cell lysates and precipitated proteins were analyzed by Western blotting and quantified by densitometry, as described above. Precipitated (active) RhoA was normalized using the corresponding total cell lysates. Data in each independent experiment were expressed as fold change from the control (taken as unity).

### 2.10 GEF activation assay

GEF activation was detected using affinity precipitation with GST- RhoA (G17A), a kind gift from Dr Keith Burridge (University of North Carolina, Chappel Hill) as described in (Dan et al., 2019; Venugopal et al., 2024; Waheed et al., 2013). The pGEX-4T1-RhoA G17A plasmid is available from Addgene. Confluent HaCaT or HEKa cells were lysed in GEF assay buffer (20 mM HEPES (pH 7.5), 150 mM NaCl, 5 mM MgCl_2_, 1% TX-100, 1 mM DTT and 1 mM PMSF and protease inhibitors), centrifuged, and an aliquot for total sample was taken. The supernatants were incubated with 25 µg of GST-RhoA (G17A) at 4°C for 45 min. Following extensive washes, the beads were pelleted. GEF-H1 activation was followed using Western blotting, as earlier (Dan et al., 2019; Venugopal et al., 2024). Aliquots for determining total GEF were taken prior to the addition of the GST-RhoA (G17A) beads. Western blots were quantified by densitometry as described above. Precipitated (active) GEF-H1 was normalized using the corresponding total cell lysates. Data in each independent experiment were expressed as fold change from the control (taken as unity).

### 2.11 RT-PCR

HaCaT cells were treated with TNFα as indicated, then RNA was extracted using the RNeasy kit (Qiagen, Valencia, CA) and cDNA was synthesized from 1 µg total RNA using iScript reverse transcriptase (Bio-Rad Laboratories). SYBR green-based real-time PCR was performed to evaluate mRNA expression of VEGF-A-165 isoform a, using GAPDH as the reference standard. Primer pairs, obtained from Invitrogen, were as follows:

Human VEGF-A-165a primers:

forward primer *5′- GAGCAAGACAAGAAAATCCC-3′*

reverse primer 5′- CCTCGGCTTGTCACATCTG-3′

Human GAPDH primers:

forward primer*: 5′-GTCTCCTCTGACTTCAACAGCG-3′*

reverse primer: 5′-ACCACCCTGTTGCTGTAGCCAA-3′

### 2.12 Immunofluorescence

Cells were grown to confluence on glass coverslips within 4-well Ibidi culture-insets, and migration was initiated by removing the chamber. At this time cells were treated as indicated in the figure legends. The cells were allowed to migrate for 6 h, then they were fixed using 4% paraformaldehyde, washed with phosphate-buffered saline (PBS), permeabilized using 0.1% Triton X100 and then blocked with 3% BSA in PBS. Next, the coverslips were incubated with primary antibody (1:100) for 1 h, then washed and bound antibody was detected using Alexa Fluor555 labelled secondary antibody (1:1,000). Nuclei were counterstained with Dapi. The slides were mounted using Dako Fluorescence Mounting Media (Agilent, Cat# S3023). Slides were visualized using a Zeiss LSM700 confocal microscopy system (40x non-oil objective). Z-stacks were obtained, and maximal intensity projection pictures were generated using the ZenBlue software. To quantify the fluorescent signal, the images were imported into ImageJ (Fiji). Each image was split into separate color channels. A Gaussian blur filter was applied with a sigma radius of 2 pixels to reduce noise and improve signal detection. A threshold of 1,400 was applied to each image to segment fluorescent signal from background. Regions of interest (ROIs) corresponding to the cell “sheet” layer were selected manually using the Freehand selection tool. Mean fluorescence intensity within each ROI was measured using the “Measure” function, with settings adjusted to record mean grey value.

### 2.13 Mouse model and immunohistochemistry (IHC)

Murine tissue samples were a kind gift from Noa Therapeutics. The MC903-induced model of atopic dermatitis (AD) (Li et al., 2009; Alam et al., 2023) was executed by TransBIOTech (Levis, QC, Canada). TransBIOTech animal care facility is accredited by the Canadian Council on Animal Care (CCAC). This study was approved by the Cégep de Lévis Animal Care Committee and complied with CACC standards and regulations governing the use of animals for research. In C57Bl/6 female mice AD was induced by daily application of calcipotriol (MC903) to the dorsal face of both ears for 6 days. Cohort Size was 3 animals/group. Mice were euthanized on Day 7 by exsanguination under isoflurane anesthesia. The upper half of the left ear was collected and fixed in formaldehyde, and embedded in 4% paraffin at 4°C. For IHC, skin sections were deparaffinized in xylene followed by rehydration using a graded series of alcohol washes. Antigen retrieval was performed by boiling sections in 10 mM sodium citrate (pH 6.0) for 20 min, followed by cooling for 20 min. Endogenous peroxide was blocked for 30 min using 3% H_2_O_2_. Nonspecific IgG binding was blocked by 2% goat serum (Cat#MP-7451) (Vectorlabs (Newark, CA). Sections were incubated with antibodies detecting pS886-GEF-H1 (1:300), or active RhoA (RhoA-GTP) (1:500), or phospho-VEGFR2 (KDR) (1:500) overnight at 4°C. Next, sections were incubated with ImmPRESS HRP goat anti-rabbit IgG (Vectorlabs, Cat# MP-7451 and MP-7452) for 30 min, and developed using a ImmPACT DAB peroxidase substrate kit (Vectorlabs, SK-4105). The tissues where counterstained with SelecTech Hematoxylin 560 (Leica Biosystems Cat# 3801575). The negative control was stained the same way, but the primary antibody was omitted. Histological slides were imaged on an Olympus BX50 microscope.

### 2.14 Statistical analysis

All experiments were performed with the number of independent experiments indicated in the figure legends. Graphs show mean +/- SD. Statistical analysis was performed using GraphPad Prism (version 10.2.3). For comparing data normalized to the control, taken as unity, we used one-sample t-test, with 1 as the hypothetical value, and used * symbols to denote significance. For comparing non-normalized samples, one-way ANOVA was used, and significance was denoted using #. Significance is indicated on the figures as follows: one symbol (# or *) p < 0.05; two symbols (## or **) p < 0.01; three symbols (### or ***) p < 0.001; four symbols (#### or ****) p < 0.0001.

## 3 Results

### 3.1 The inflammatory cytokine TNFα induces VEGF synthesis and release in keratinocytes

Keratinocytes are a rich source of inflammatory mediators that have important regenerative paracrine effects. However, much less is known about how these keratinocyte-derived soluble factors affect keratinocyte biology via autocrine effects. To address this gap, we first sought to identify mediators that were induced by the inflammatory cytokine TNFα. We collected conditioned media from HaCat keratinocytes that were unstimulated or exposed to TNFα for 16 h and analyzed the secretome using a Human Cytokine/Chemokine 48-Plex Discovery Assay array (Eve Technologies). Figure 1A shows results from 3 independent repeats. Only cytokines that were in the readable range are depicted. Positive z-scores indicate values above the mean, while negative z-scores indicate values below the mean. Unstimulated cells (left 3 columns) produced a range of cytokines and growth factors, with some variations in the basal expression across the three repeats. Simultaneous detection of multiple factors revealed that TNFα increased secretion of 35 factors (Figure 1A, compare pairwise the left and right three columns). Figure 1B shows the fold change in these mediators. Several chemokines were strongly upregulated, including MIG/CXCL9, monocyte chemoattractant protein 1 (MCP1, also known as CCL2), Growth-regulated Oncogene 1 (GROα or CXCL1), MDC (CCL22) and fractalkine (CX3CL1), although due to the variability of the effect only the change in fractalkine reached statistical significance. Several interleukins and interferons were also consistently increased, including IL-2, 6, 15 and 17. Growth factors stimulated by TNFα included Transforming Growth Factor (TGF) α and β and Vascular Endothelial Growth Factor (VEGF)-A. Of these, the increase in VEGF-A was statistically significant (Figures 1A–C). Since VEGF-A and especially the VEGF-165 isoform has strong effects on cell migration (Takahashi and Shibuya, 2005), we decided to further study this growth factor. We used an ELISA to assess the time-dependent increase in VEGF-A-165 isoform. HaCat cells continuously released VEGF-165 (Figure 1D), as indicated by its accumulation over time in the conditioned media. Importantly, VEGF-165 release was significantly augmented by TNFα as soon as 3 h after stimulation and further increased at all timepoints studied. VEGF-165 also increased in primary adult normal keratinocytes (HEKa cells) (Figure 1E). TNFα also significantly elevated the mRNA of the VEGF-165a isoform in HaCat cells (Figure 1F), indicating increased synthesis. Indeed, *de novo* synthesis was essential for TNFα-induced VEGF-165 release, as Actinomycin D, an RNA polymerase inhibitor, eliminated the effect (Figure 1G). In fact, VEGF-165 release in Actinomycin D-treated cells dropped below the unstimulated levels verifying continuous synthesis of VEGF-165. In endothelial cells and embryonic tissues HIF-1α and SP1 were shown to be key for stimulating the VEGF-A promoter (Pagès and Pouysségur, 2005), and in macrophages and ovarian cancer cells, VEGF synthesis was also found to be partly NFκB-dependent (Kiriakidis et al., 2003; Huang et al., 2000). Therefore, we next assessed the role of these transcription factors using specific inhibitors. Figure 1H shows that Echinomycin (HIF-1α inhibitor), Mithramycin-A (SP1 inhibitor), and Bay11-7085 (NFκB inhibitor) significantly reduced basal VEGF-165 release and prevented the TNFα-induced increase. Importantly, neither Echinomycin nor Mithramycin-A alone or in combination with TNFα affected cell viability (Supplementary Figure S1). In contrast, the NFκB inhibitor significantly reduced cell viability, in line with previous reports indicating that keratinocyte survival is dependent on NFκB (Qin et al., 2001). However, the NFκB inhibitor prevented the VEGF release fully, but reduced viability only about 50%, thus it is likely that NFκB was also necessary for the VEGF-inducing effect of TNFα. Taken together, TNFα stimulates the release of various factors in keratinocytes, including VEGF-165, that is augmented through HIF1α, SP1 and likely NFκB.

### 3.2 TNFα enhances keratinocyte migration through VEGF-A

**FIGURE 2:**
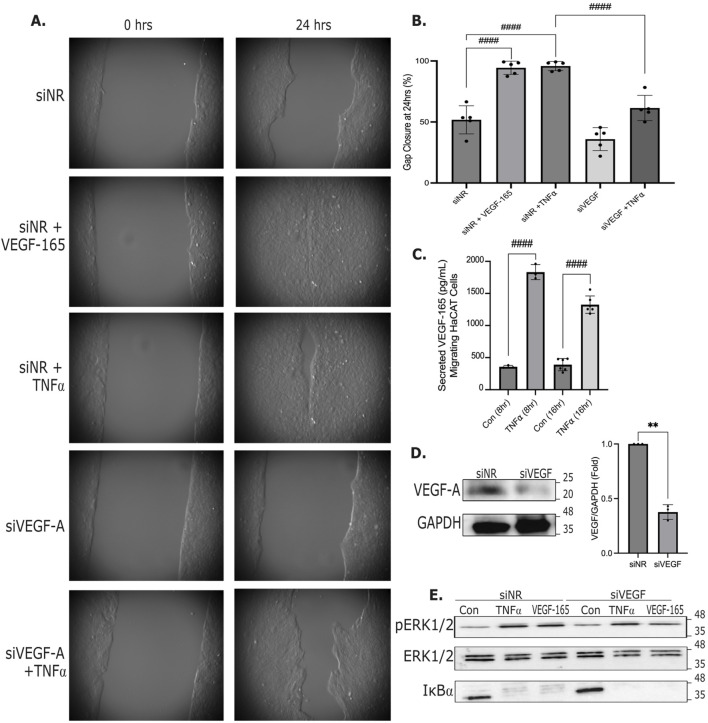
TNFα augments keratinocyte migration through VEGF-A. **(A)** HaCat cells were transfected with control (non-related, NR) or VEGF-A siRNA (100 nM), and cell migration was followed as described in the Methods. Where indicated, VEGF-165 (100 ng/mL) or TNFα (20 ng/mL) was added at the initiation of migration. Representative pictures taken at the indicated times are shown. **(B)** Gap closure was analyzed using the T-scratch software and % gap closure was calculated and plotted for the 24 h timepoint (n = 5, one-way ANOVA: ####p < 0.0001). **(C)** VEGF-165 was measured as in Figure 1C in conditioned media of migrating cells at 8 h and 16 h. Where indicated, cells were treated with TNFα (n = 3 (8 h timepoint) and n = 6 (16 h timepoint), one-way ANOVA: ####p < 0.0001). **(D)** Cells were transfected with NR of VEGF-A siRNA for 24 h. VEGF-A and GAPDH were detected using Western blotting. The graph shows densitometry analysis. VEGF-A levels were normalized to GAPDH and expressed as fold change from the control taken as unity. (n = 3, one sample t-test vs. 1: **p < 0.01). **(E)** Cells were transfected with NR of VEGF-A siRNA as in D, and 24 h later cells were stimulated with VEGF-165 or TNFα for 15 min. Levels of phospho-ERK1/2, total ERK1/2 and IκBα were detected by Western blotting as in D. Original uncropped blots are shown in Supplementary Figure S2.

Since VEGF-165 is a potent pro-migratory factor, we next tested its role in keratinocyte migration, using live imaging. HaCat cells were grown on glass bottom plates in Ibidi chambers with a divider in the middle to generate a gap in the layer. Migration into the gap was initiated by removing the chamber. Migrating cells were imaged for 24 h, and closure of the gap was quantified using the T-scratch software. As shown on Figure 2A, HaCat cells migrate in a sheet causing about 50%–60% gap closure by 24 h. Addition of 100 ng/mL VEGF-165 potently enhanced migration, reducing the time of gap closure to about 16 h, and causing complete closure of the gap by 24 h (Figures 2A,B). TNFα (20 ng/mL) caused a similar increase in migration, leading to complete or near complete closure at 24 h (Figures 2A,B). Migrating layers also released a well detectable amount of VEGF-165, augmented by TNFα (Figure 2C). This raised the possibility that VEGF-165 may have a role as an autocrine mediator enhancing migration. To test this, we transfected cells with a VEGF-A-specific siRNA, that induced a strong reduction in VEGF-A levels (Figure 2D). VEGF-A depletion significantly slowed basal cell migration and blunted the effect of TNFα (Figures 2A,B). Both VEGF-165 and TNFα potently augmented ERK1/2 phosphorylation and IκBα degradation, indicating activation of ERK and the NFκB pathways (Figure 2E). These effects were not affected by VEGF-A silencing, revealing that not all effects of TNFα were mediated by VEGF-A and the TNF receptor remained activatable in the absence of VEGF-A.

### 3.3 Pro-migratory effects of VEGF-A and TNFα are mediated by KDR

**FIGURE 3:**
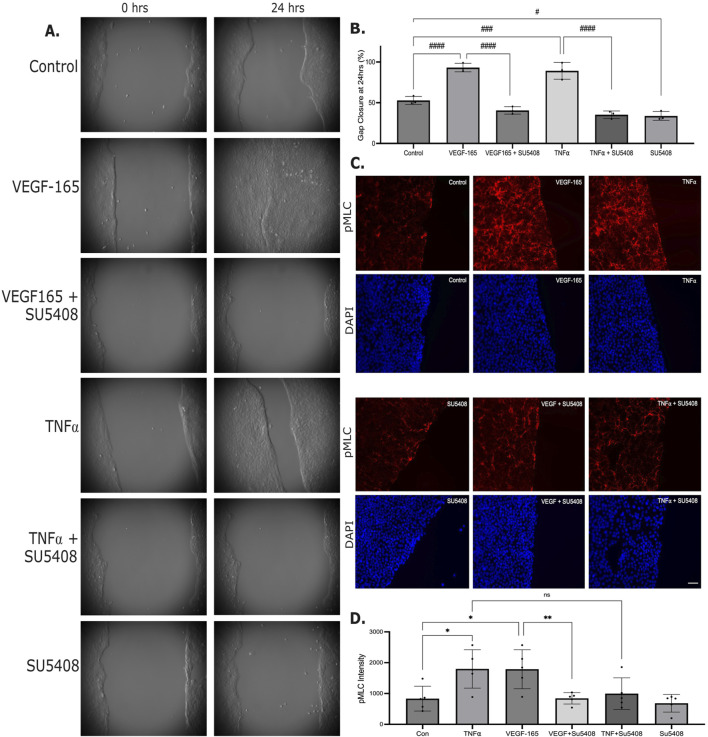
KDR is essential for migration. **(A,B)** Cell migration was measured as in Figure 2. Cells were treated with VEGF-165 (100 ng/mL) or TNFα (20 ng/mL) or SU5408 (100 nM), as indicated. A shows representative images at the indicated times, B shows quantification done as in Figure 2 (n = 3, One-way ANOVA, #p < 0.05, ##p < 0.01, ####p < 0.0001). **(C)** Cells were grown on glass coverslips within 4-well Ibidi culture-insets. At confluence, cells were treated with VEGF-165 or TNFα with or without SU5408, and migration was initiated. Cells were fixed 6 h later, and phospho-MLC (top rows, red) and nuclei (DAPI staining, bottom rows, blue) were detected. Representative z stacks are shown. The size bar on the bottom right image represents 10 µm and applies to all images. **(D)** Quantification of the pMLC fluorescent signal was done as described in the Methods. n = 5 one-way ANOVA, *p < 0.05, **p < 0.01).

In endothelial cells VEGF-A was shown to augment migration through VEGFR2/KDR (Zeng et al., 2002). To test the role of this receptor (referred to as KDR), we first used SU5408, the most potent and selective KDR inhibitor among the new 3-substituted indolin-2-ones group of inhibitors (Sun et al., 1998). As shown on Figures 3A,B, SU5408 significantly reduced basal migration and abrogated the effects of externally added VEGF-165. Importantly, the inhibitor also prevented the effect of TNFα, again implying a role for KDR in migration.

Since acto-myosin contractility is crucial for cell migration, next we tested whether VEGF-165 and TNFα augmented myosin light chain (MLC) phosphorylation in the migrating layer. Six hours after initiating the migration, the control layers showed some pMLC staining, which was significantly augmented by VEGF-165 and TNFα (Figures 3C,D). SU5408 strongly reduced pMLC increase induced by VEGF-165 and TNFα, although quantification revealed that the inhibition of the TNFα effect did not reach statistical significance.

**FIGURE 4:**
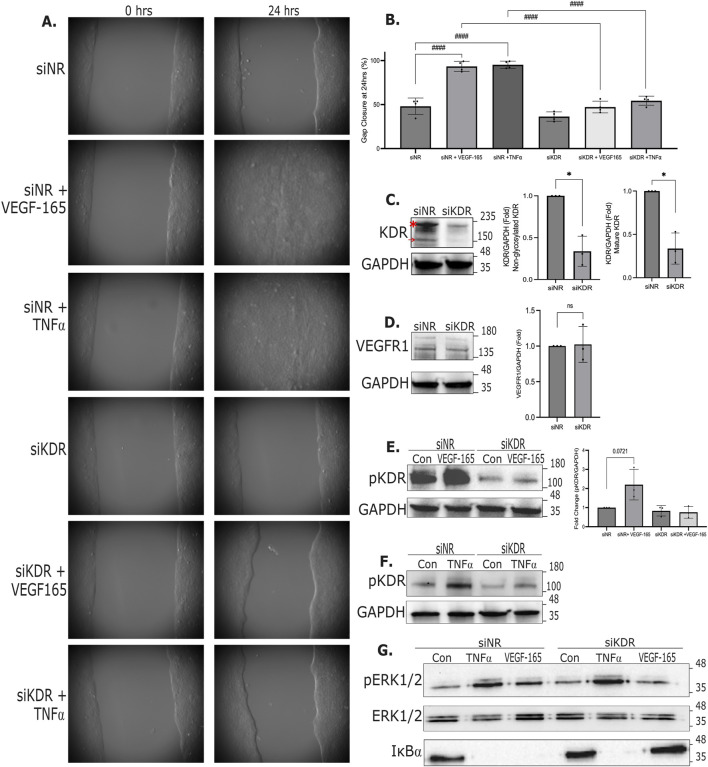
VEGF-A and TNFα-induced migration is inhibited by KDR silencing. **(A,B)** HaCaT cells were transfected with NR or KDR siRNA, and migration was followed as in Figure 2. Where indicated, cells were treated with VEGF-165 or TNFα. In A, representative images are shown. In B gap closure was analyzed as above. Please note, the control (siNR), TNFα- and VEGF-165-treated groups are a subset of those shown on Figure 2B, since some of the VEGF and KDR silencing experiments were done simultaneously with the same controls (n = 4, One-way ANOVA, ####p < 0.0001). **(C–G)** Cells were transfected with NR of KDR-specific siRNA. In **(E-G)** cells were treated with VEGF-165 or TNFα for 15 min. Levels of KDR, VEGFR1, pKDR and pERK1/2, ERK1/2 and IκBα were detected by Western blotting as in Figure 2. (n = 3, one sample t-test vs. 1 *p < 0.05). In C the red asterisk indicates the glycosylated (mature) form of KDR, and the arrow points to the non-glycosylated version. Original uncropped blots are shown in Supplementary Figure S3.

To further corroborate the role of KDR in cell migration, we next silenced this protein using a specific siRNA. Consistent with the effect of the inhibitor, KDR silencing also impaired migration (Figures 4A,B). Efficient silencing of both the mature KDR (a transmembrane glycoprotein with a molecular weight above 200 kDa, red asterisk) and the non-glycosylated form (150 kDa, red arrow) was verified using Western blotting (Figure 4C). (Wang et al., 2020) Importantly, the KDR siRNA did not alter the expression of VEGFR1 (Figure 4D). KDR siRNA also efficiently reduced the VEGF-induced changes in phospho-KDR (Figure 4E). As shown on Figure 4F, TNFα increased phospho-KDR, indicating transactivation of KDR. Interestingly, while the effects of VEGF-165 on ERK1/2 phosphorylation and IκBα degradation were abolished by KDR depletion, the TNFα-induced ERK1/2 phosphorylation and IκBα degradation were not affected (Figure 4G). This corroborated KDR-independent effect of TNFα and showed that the TNF receptor remained able to signal when KDR was silenced. Taken together, these data show that VEGF-165 is a cell autonomous autocrine mediator augmenting migration through KDR. VEGF-165 and KDR are indispensable for the TNFα-induced stimulation of migration.

### 3.4 VEGF-A stimulates GEF-H1 through KDR and ERK

**FIGURE 5:**
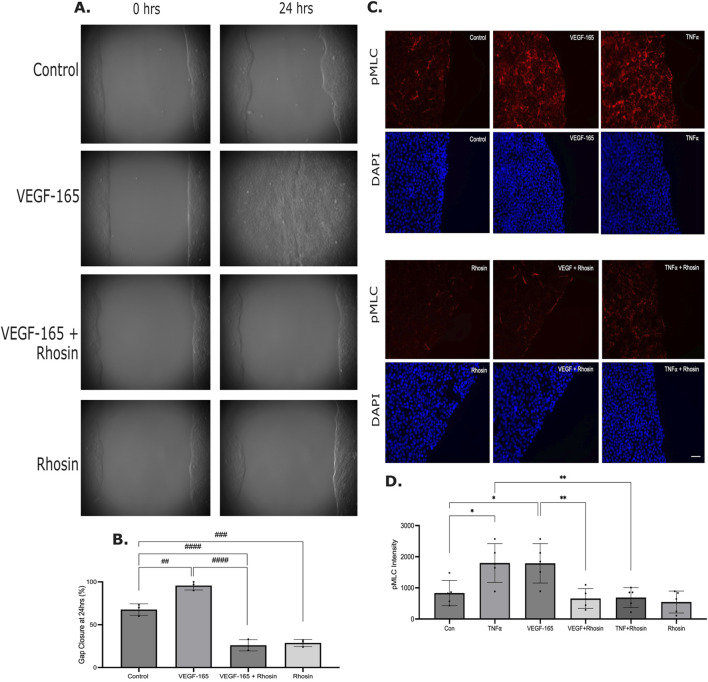
RhoA is essential for keratinocyte migration and pMLC increase. **(A,B)** Migration was followed as in Figure 2. Where indicated, cells were treated with VEGF-165 and/or Rhosin (30 µM) (n = 3, One-way ANOVA: ##p < 0.01; ###p < 0.001, ####p < 0.0001). **(C)** pMLC was detected in migrating cells as in Figure 3. Where indicated, cells were treated with Rhosin, or VEGF-165 or TNFα in the absence or presence of Rhosin. Representative z stacks are shown. The size bar on the bottom right image represents 10 µm and applies to all images. **(D)** Quantification of the fluorescent signal was done as described in the Methods. n = 5, one-way ANOVA, *p < 0.05, **p < 0.01).

Small GTPases are essential regulators of actin remodeling and pMLC during cell migration (Machacek et al., 2009; Zegers and Friedl, 2014) and VEGF-A-induced RhoA activation is important for endothelial cell migration and angiogenesis (Van Nieuw Amerongen et al., 2003). Indeed, using a RhoA-specific inhibitor, Rhosin, we found significantly reduced gap closure in HaCaT cells both with and without VEGF-165 addition (Figures 5A,B). In line with the crucial role of RhoA/Rho kinase in MLC phosphorylation, Rhosin also significantly reduced basal pMLC, as well as VEGF-165 or TNFα-induced increase in pMLC (Figures 5C,D).

**FIGURE 6:**
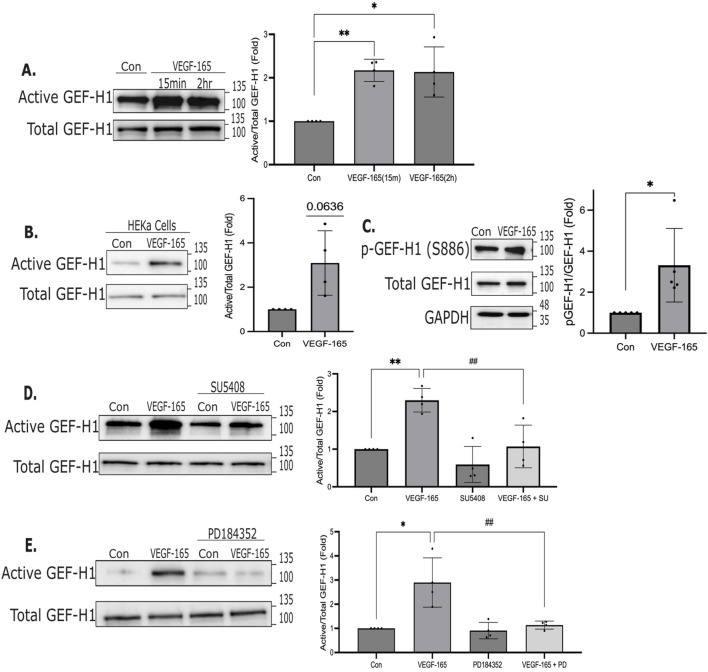
GEF-H1 is activated by VEGF-A. **(A)** HaCaT cells were treated with VEGF-165 for 15 min or 2 h. Activated GEFs were precipitated using RhoA (G17A). GEF-H1 in the precipitates (active) and in the total cell lysates (total) was detected and quantified using Western blotting and densitometry. Active GEF-H1 was normalized with the corresponding total GEF-H1 and expressed as fold change from the control taken as unity (n = 4, one sample t-test vs. 1: *p < 0.05, **p < 0.01). **(B)** HEKa cells were treated with VEGF-165 and GEF-H1 activity was assessed as above. n = 4, one sample t-test. **(C)** HaCat cells were treated for 15 mins with VEGF-165, and phospho-S886-GEF-H1, total GEF-H1 and GAPDH were quantified using Western blotting. pS886-GEF-H1 levels were normalized with the corresponding total GEF-H1 and expressed as fold change from control, taken as unity. (n = 5, one sample t-test vs. control *p < 0.05). **(D,E)** HaCat cells were pre-treated with SU5408 (30 min) **(D)** or PD184352 (15 min) 10 μM **(E)** and subsequently treated with VEGF-165 for 15 mins with or without the inhibitors. Active GEF-H1 was precipitated and quantified as in A. (n = 4, one sample t-test vs. 1 *p < 0.05, **p < 0.01; and one-way ANOVA: ##p < 0.01). Original uncropped blots are shown in Supplementary Figure S4.

The GEF(s) mediating VEGF-induced RhoA activation remain poorly studied. Recently, GEF-H1 (ArhGEF2) was suggested to control cortical RhoA activity in keratinocytes (Müller et al., 2024; Nalbant et al., 2009). Since we have previously shown that GEF-H1 was essential for migration in tubular cells (Waheed et al., 2013), we decided to focus on this GEF. To test whether GEF-H1 was activated by VEGF-165, we used an affinity precipitation assay, as in previous studies (Dan et al., 2019; Venugopal et al., 2024; Waheed et al., 2012). This assay takes advantage of GST-RhoA (G17A), a mutant that is unable to bind nucleotides, and has high affinity towards activated GEFs. Control and VEGF-165-stimulated cells were lysed, and GEFs were precipitated using GST-RhoA (G17A). Precipitated proteins were analyzed using Western blotting to detect GEF-H1. As shown in Figure 6A, in HaCat cells VEGF-165 induced a significant activation of GEF-H1 both at 15 min and 2 h. VEGF-165 also activated GEF-H1 in primary keratinocytes (Figure 6B). Further, VEGF-165 also elevated phosphorylation of GEF-H1 at the S886 site (Figure 6C). SU5408 prevented VEGF-165-induced GEF-H1 activation, indicating a role for KDR (Figure 6D). Since VEGF-165 potently activated ERK1/2 through KDR (Figure 4G), and ERK was shown to promote GEF-H1 activation (Kakiashvili et al., 2011), we asked if VEGF-165 activated GEF-H1 through ERK. Indeed, the MEK1/2 inhibitor PD184352 reduced VEGF-165-induced GEF-H1 activation (Figure 6E). Thus, VEGF-165 is a potent activator of GEF-H1 through KDR and ERK1/2.

### 3.5 GEF-H1 is essential for keratinocyte migration and VEGF-A production

**FIGURE 7:**
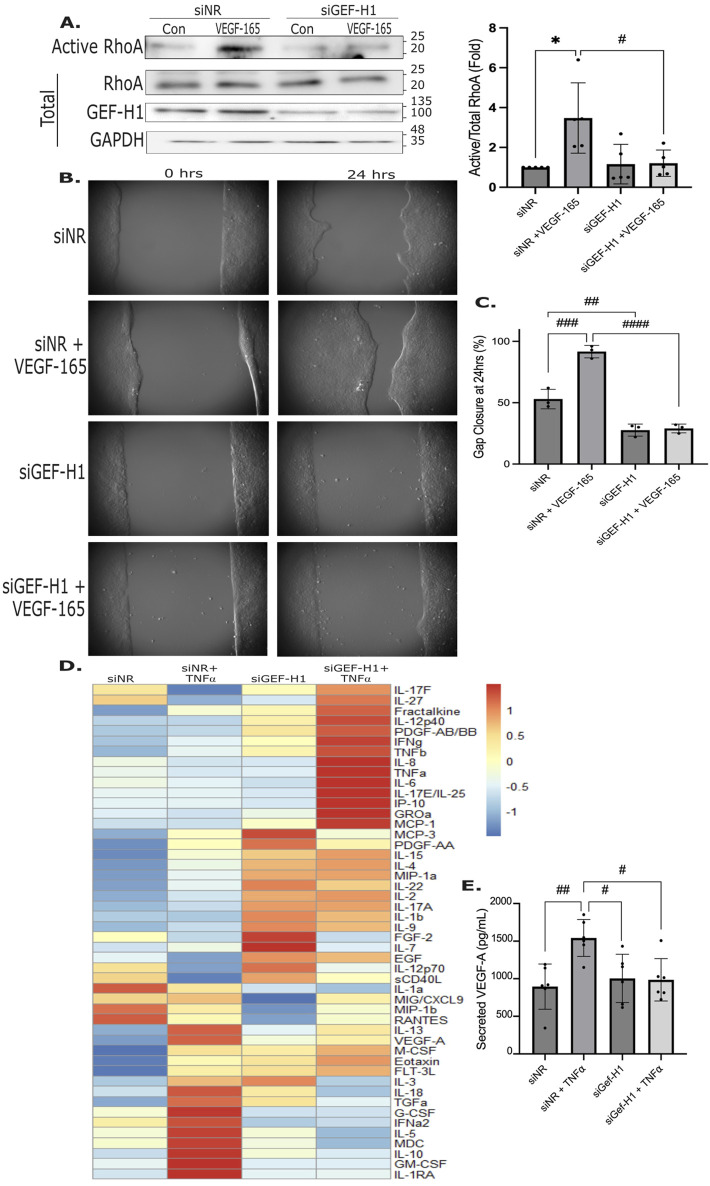
Role of GEF-H1 in VEGF-induced RhoA activation and keratinocyte migration. **(A)** HaCaT cells were transfected with NR or GEF-H1 siRNA (100 nM) for 48 h. Where indicated, cells were treated with VEGF-165 (15 min). Cells were lysed, and active RhoA was captured using GST-RBD. RhoA was detected in the precipitates (active RhoA) and RhoA, GEF-H1 and GAPDH were detected in the total cell lysates. Densitometry values for active RhoA in each sample were normalized using total RhoA and expressed as fold change from control taken as unity (n = 5, one sample t-test vs. 1 *p < 0.05; one-way ANOVA: #p < 0.05). **(B,C)** HaCaT cells were transfected with NR or GEF-H1-specific siRNA, and migration was measured as in Figure 2. Where indicated, VEGF-165 was added to the cells at the initiation of migration (n = 3, one-way ANOVA: ##p < 0.01, ###p < 0.001, ####p < 0.0001). **(D,E)** HaCaT cells were transfected with NR or GEF-H1 siRNA for 24 h, then treated with TNFα for 24 h. In **(D)** the secretome was analyzed by a multiplex assay as in Figure 1, z-scores were calculated using the average of 3 independent experiments and depicted in the heatmap. In **(E)** VEGF-165 release was measured using an ELISA as in Figure 1 (n = 6, one-way ANOVA: #p < 0.05, ##p < 0.01). Original uncropped blots are shown in Supplementary Figure S5.

To assess the role of GEF-H1 in VEGF-165-induced RhoA activation, we depleted this GEF using a specific siRNA, resulting in strong reduction in GEF-H1 levels (Figure 7A). VEGF-165 induced RhoA activation, that was prevented by silencing GEF-H1 (Figure 7A). Next, we assessed the effect of GEF-H1 depletion on cell migration. GEF-H1 knockdown significantly impaired both basal and VEGF-165 stimulated cell migration, as shown by reduced gap closure (Figures 7B,C), indicating that this GEF is essential for migration.

Since GEF-H1 was suggested to promote cytokine production in macrophages, we next asked if GEF-H1 knockdown might affect the TNFα-induced secretome. Conditioned media from cells transfected with a control or a GEF-H1-specific siRNA with or without stimulation with TNFα were analyzed by the Cytokine/Chemokine 48-Plex Discovery Assay Array. Figure 7D shows average change in 3 independent repeats/conditions. GEF-H1 depletion reduced the TNFα-induced release of a group of mediators, including VEGF-A, IL5, IL10, MDC, IL13, IL18, and TGFα (Figure 7D). Interestingly, silencing GEF-H1 by itself potently augmented a group of cytokines, including IL22, IL17, IL1b, Eotaxin, and PDGF-AA. For a third group, including TNFα itself, GEF-H1 depletion augmented the stimulatory effect of TNFα. The effect of GEF-H1 silencing on VEGF-165 was verified using the VEGF-165 ELISA kit. As shown on Figure 7E, the TNFα-induced VEGF-165 release was blunted by downregulation of GEF-H1, suggesting a feedback regulation.

### 3.6 GEF-H1, phospho-KDR, and Rho-GTP are upregulated in an inflammatory mouse model

**FIGURE 8:**
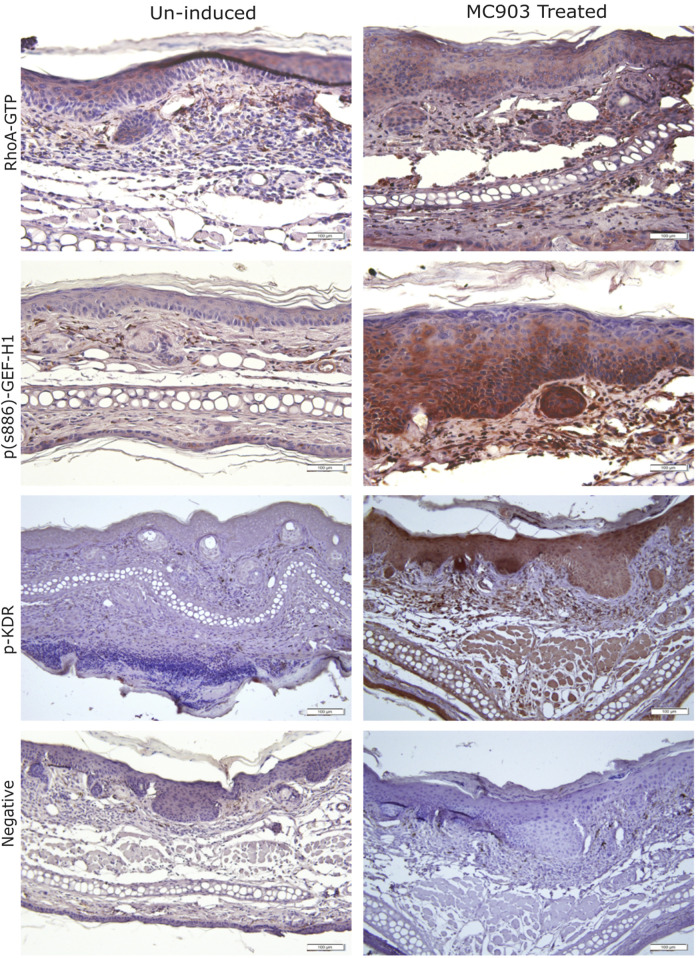
Activation of the KDR/GEF-H1/RhoA pathway in a murine atopic dermatitis model. Skin samples from the ear of untreated mice, or mice where AD-like inflammation was induced using calcipotriol (MC903). Representative images show RhoA-GTP, phospho-KDR or phospho-S886GEF-H1 staining (brown). The tissues were counterstained with H&E. The negative control was processed the same way, without primary antibody.

Our findings imply that VEGF-A affects keratinocytes through KDR and GEF-H1/RhoA. To substantiate that these are indeed activated during skin inflammation in the epiderims, we tested samples from a widely used AD mouse model (Li et al., 2009; Alam et al., 2023). Application of calcipotriol (MC903) to the ears of mice for 6 days induces an atopic dermatitis-like skin inflammation. To assess changes in KDR, GEF-H1 and RhoA, we performed immunohistochemistry analysis using specific antibodies detecting active versions of these proteins. Our data revealed that skin inflammation indeed elevated levels of phospho-S886 GEF-H1, phospho-KDR, and RhoA-GTP (i.e., active RhoA) compared to untreated controls (Figure 8). These findings suggest that the KDR/GEF-H1/RhoA signaling axis is active in inflammatory skin conditions, such as atopic dermatitis.

## 4 Discussion

**FIGURE 9:**
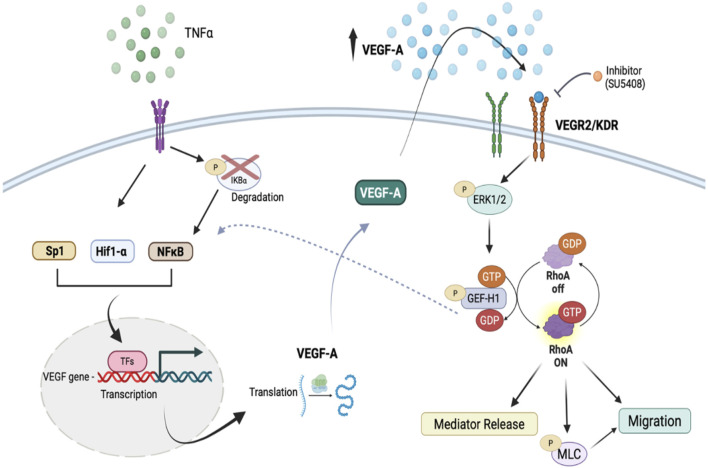
A schematic showing the proposed mechanism whereby TNFα promotes VEGF release and activates migration. Created in BioRender. https://BioRender.com/yhnys5s.

In this study we demonstrated a key autocrine role for VEGF-165 in keratinocyte migration, acting through KDR and GEF-H1/RhoA signalling. We also showed that TNFα induced VEGF-165 synthesis and release, and transactivation of KDR, that are crucial for augmented migration. The proposed mechanisms are summarized in Figure 9.

### 4.1 TNFα is a potent inducer of VEGF-A synthesis and release

Compelling evidence shows that TNFα is crucial for skin homeostasis and for responses to environmental damage, such as ultraviolet B (UVB) light (Bashir et al., 2009a; Bashir et al., 2009b) and skin infection (Köck et al., 1990; Aufiero et al., 2007). TNFα upregulates genes related to immune responses, inflammation, cell cycle, survival and migration in keratinocytes (Banno et al., 2004). Our data showing simultaneous increase in an array of cytokines, chemokines and growth factors, including VEGF-A, substantiate the role of TNFα as a master regulator of skin immune responses and tissue regeneration.

The human VEGF family includes several members with different functions (reviewed in (Takahashi and Shibuya, 2005)). We found strong upregulation of VEGF-A in both HaCaT cells and primary keratinocytes. Human epidermal keratinocytes can synthesize three isoforms of VEGF-A protein (VEGF 189, 165, and 121) (Ballaun et al., 1995; Detmar et al., 1995; Brown et al., 1992) which are elevated by growth factors and cytokines, including TNFα (Frank et al., 1995; Longuet-Perret et al., 1998). VEGF-165 was described as the predominant isoform (Takahashi and Shibuya, 2005), that in endothelial cells has promigratory and angiogenic effects (Van Nieuw Amerongen et al., 2003). It was also shown to modulate skin regeneration in ischemic wounds (Huang et al., 2012). Our study verified an increase in VEGF-165 upon TNFα treatment in both HaCaT cells and primary keratinocytes. Future studies should clarify changes in other isoforms, and their potential effects on keratinocyte functions. TNFα increased VEGF-165 in keratinocytes through HIF1α, SP1 and NFκB, similar to the mechanism described in endothelial cells (Martin et al., 2009). This finding is also in line with a report showing that in HaCaT cells UVB light-induced) VEGF-165 up-regulation through HIF1α and AP2/SP1 (Wunderlich et al., 2008; Brenneisen et al., 2003). HIF1α and SP1 likely directly control the VEGF-A promoter, although we have not tested this, and an indirect effect cannot be ruled out. A possible direct promoter-inducing effect of NFκB is less clear, since its inhibition reduced cell viability, indicating it may have an indirect effect. Taken together, our study supports a general role of TNFα as a regulator of VEGF-165 in keratinocytes, integrating effects of a multitude of physiological and pathological stimuli.

### 4.2 VEGF-A is an autocrine mediator acting through KDR

We found that both HaCaT cells and primary keratinocytes continuously released VEGF-165, that acted as an autocrine mediator, essential for both basal and TNFα-induced keratinocyte migration. A VEGF-A/KDR autocrine loop promoting keratinocyte migration and mediator release may be important in homeostasis but could also represent an early response to damage. Although keratinocyte-derived paracrine effects are well described, potential autocrine regulation has been less explored. Keratinocyte-derived TGFα, an EGF receptor activator, was shown to augment migration (Ju et al., 1993), and enhance normal and malignant keratinocyte growth via an autocrine loop (Partridge et al., 1989). In various cancer cells autocrine VEGF-A effects were linked to proliferation (Wang et al., 2013; Perrot-Applanat and Di Benedetto, 2012). In skin carcinoma, IL-6-induced VEGF-A contributes to tumor growth in an autocrine loop (Lederle et al., 2011). Our studies extend such findings by implying a key role for a VEGF autocrine effect in keratinocyte migration. During the complex skin inflammatory responses other cell types, such as Langerhans cells, immune cells, endothelial cells and fibroblasts are also a major source of VEGF-A, likely affecting keratinocytes. Keratinocyte-derived VEGF-165 also has crucial paracrine effects, e.g., on dermal endothelial cells and angiogenesis (Detmar et al., 1995; Detmar et al., 1998).

### 4.3 TNFα transactivates KDR

Our studies revealed transactivation of KDR by TNFα that is key for efficient migration. Indeed, TNFα is known to transactivate various growth factor receptors in many cells. For example, our earlier studies have shown that in tubular cells TNFα activated GEF-H1 through EGF receptor transactivation (Kakiashvili et al., 2011). Thus, the outcome of TNFα stimulation in keratinocytes is likely a combination of direct TNF receptor (TNFR)-induced signalling, and transactivation of other receptors, including KDR. The types of VEGF receptors expressed in keratinocytes remain debated. Some studies reported that in murine keratinocytes only VEGFR1 was present (Wilgus et al., 2005; Benhadou et al., 2020). Epidermal autonomous functions of Flt1 (VEGFR1) were found to be essential for VEGF-A-induced psoriasis-like disease in mice (Benhadou et al., 2020). In contrast, functional KDR is present in the human epidermis (Man et al., 2006) and was found to be overexpressed in psoriatic epidermis (Man et al., 2008). UV also upregulated KDR in normal and psoriatic human keratinocytes (Zhu et al., 2020), and was suggested to mediate pro-survival effects following moderate dose UV irradiation (Zhu et al., 2012). Our experiments using a KDR inhibitor and siRNA-mediated KDR silencing revealed essential roles for this receptor in migration and GEF-H1 activation. Although we have not specifically explored the role of VEGFR1, KDR silencing did not affect the expression of VEGFR1, supporting a role for KDR and not VEGFR1.

### 4.4 GEF-H1/RhoA activation by VEGF-A is essential for migration

Our experiments verified that RhoA inhibition prevented keratinocyte migration. RhoA has emerged as an essential regulator of keratinocyte functions beyond migration. It controls the balance between proliferation and differentiation in cultured basal keratinocytes by coordinating adhesion, cytoskeletal organization, and cell cycle exit (McMullan et al., 2003; Tu et al., 2011; Calautti et al., 2002). Contrasting these findings, in a keratinocyte-specific KO animal RhoA showed no changes in skin development (Jackson et al., 2011). While the reason for this discrepancy remains unclear, interesting studies suggest that different Rho effectors have opposite effects on terminal differentiation. Rho kinase (ROCK)1 was shown to promote differentiation, while ROCK2 and citron kinase inhibited it (McMullan et al., 2003; Grossi et al., 2005; Calautti et al., 2002; Lock and Hotchin, 2009). Irrespective of these intriguing findings, Rho kinase inhibitors were found to promote proliferation and extend life-span of cultured keratinocytes and are beneficial for cultured skin substitutes (Chapman et al., 2014; M et al., 2022; Chapman et al., 2010).

We identified GEF-H1 as a KDR-activated RhoA-GEF essential for keratinocyte migration. GEF-H1 is a microtubule and cell junction bound exchange factor (reviewed in (Joo and Olson, 2021; Li et al., 2024)), that was implicated in a broad range of cellular functions, including junction regulation (Benais-Pont et al., 2003; Birukova et al., 2006)^,^ cytokinesis (Birkenfeld et al., 2007), cell cycle control (Aijaz et al., 2005; Fine et al., 2016) and osmotic stress signalling (Ly et al., 2013). It promotes carcinogenesis (Mizuarai et al., 2006; Cullis et al., 2014) and fibrogenesis (Venugopal et al., 2024; Hu et al., 2023). Here we found that VEGF-165 induced GEF-H1 activation through ERK1/2. ERK has emerged as a key positive regulator of GEF-H1, directly phosphorylating it on T678 (Waheed et al., 2013; Fujishiro et al., 2008). ERK mediates activation by various input, including TNFα, membrane potential changes and mechanical stimuli (Waheed et al., 2010; Guilluy et al., 2011). Interestingly, VEGF-165 also promoted GEF-H1 S886 phosphorylation. The exact role of phosphorylation at this site remains contradictory. On the one hand, it was suggested to control binding to 14-3-3 or Tctex-1, that sequester inactive GEF-H1 to the microtubules (Meiri et al., 2014; Zenke et al., 2004; Yamahashi et al., 2011). In contrast, in several studies, including ours, increased S886 phosphorylation correlated with activation (Waheed et al., 2013; Nishida et al., 2021; He et al., 2021; Jiu et al., 2017). Concomitant phosphorylation of several sites might explain these discrepancies. The mechanisms whereby GEF-H1 affects migration could also be complex. In addition to RhoA, it was also shown to activate Rac at the leading edge (Nalbant et al., 2009; Tonami et al., 2011) and control focal adhesion turnover, in part through RhoB (Vega et al., 2012). Finally, we found that GEF-H1 also affected keratinocyte secretion. Consistent with this, RhoA and Rho kinase are crucial for UVB light-and oxidative stress-induced activation of NFκB in HaCat keratinocytes (Liang et al., 2017). GEF-H1 was also shown to contribute to pathogen-induced NFκB activation (Chang et al., 2023; Fukazawa et al., 2008; Guo et al., 2012; Zhao et al., 2012). Similar roles in keratinocyte secretion remain to be explored. Surprisingly, however, we also identified a set of inflammatory mediators that were suppressed by GEF-H1, as its depletion augmented their release. Thus, the role of GEF-H1 in keratinocyte secretion might be complex, and our future studies will further address underlying mechanisms.

### 4.5 Implications for atopic dermatitis and inflammatory skin conditions

We showed that the KDR/GEF-H1/RhoA axis was activated in a mouse AD model, where skin inflammation was induced by the active vitamin D3 analog MC-903 (calcipotriol). Previous studies in this model demonstrated elevated mRNA of several keratinocyte-derived cytokines, including VEGF-A (Liu et al., 2022). Importantly, VEGF has been implicated in several human skin diseases. The normal epidermis expresses low levels of VEGF, but its levels are elevated in psoriasis (Detmar et al., 1994), contact dermatitis (Brown et al., 1995) and skin wounds (Brown et al., 1992). Thus, KDR-mediated chronic activation of GEF-H1 and RhoA could be significant in pathology, and GEF-H1 may offer a promising therapeutic target for skin inflammation. Of note, a recent study has demonstrated that a GEF-H1 inhibitory peptide mitigated endothelial migration and reduced retina dysfunction (Mills et al., 2022). Testing the effect of such inhibitors on keratinocyte migration could be an exciting next step.

### 4.6 Limitations

Cultured keratinocytes are broadly used to uncover new regulatory mechanisms, as they allow a simplified system to manipulate proteins and detect functions. Indeed, the use of cultured keratinocytes under conditions that model proliferating basal cells allowed us to explore cell autonomous functions and probe role of specific pathways. However, such a simplified model does not recapitulate the complexity of the stratified epidermis, where keratinocyte properties differ in the basal and suprabasal layers. Interactions between keratinocytes and other cells also provide crucial input during inflammation, that our model did not capture. Further, we focused on TNFα and VEGF-A, but the inflammatory and wound healing microenvironment contains a multitude of mediators that modulate outcome. Thus, our findings, including the signaling axis we described must be validated in more physiologically relevant epidermis models and animals. Although the current study verified activation of the signalling axis, the functional role remains to be tested. Importantly, this study lays the foundation for such future mechanistic explorations and translational studies.

## 5 Conclusion

Taken together, our findings establish a key role for VEGF-A release in basal and TNFα-induced keratinocyte migration. We showed that VEGF-165 acts through KDR, and that TNFα requires VEGF-165 release and KDR transactivation to augment cell migration (Figure 9). Moreover, we identified GEF-H1 as a VEGF-stimulated GEF, the activity of which is essential for both keratinocyte migration and mediator release. This pathway could play a crucial role in normal and dysregulated wound healing and in inflammatory skin diseases, such as atopic dermatitis. Targeting key components of this signaling axis could offer new therapeutic strategies for enhancing tissue regeneration and managing skin inflammation.

## Data Availability

The raw data supporting the conclusions of this article will be made available by the authors, without undue reservation.

## References

[B1] Aijaz S. D’Atri F. Citi S. Balda M. S. Matter K. (2005). Binding of GEF-H1 to the tight junction-associated adaptor cingulin results in inhibition of Rho signaling and G1/S phase transition. Dev. Cell 8, 777–786. 10.1016/j.devcel.2005.03.003 15866167

[B2] Alam M. J. Xie L. Yap Y.-A. Robert R. (2023). A mouse model of MC903-Induced atopic dermatitis. Curr. Protoc. 3, e695. 10.1002/cpz1.695 36913546

[B3] Aufiero B. Guo M. Young C. Duanmu Z. Talwar H. Lee H. K. (2007). *Staphylococcus aureus* induces the expression of tumor necrosis factor-alpha in primary human keratinocytes. Int. J. Dermatol 46, 687–694. 10.1111/j.1365-4632.2007.03161.x 17614795

[B4] Ballaun C. Weninger W. Uthman A. Weich H. Tschachler E. (1995). Human keratinocytes express the three major splice forms of vascular endothelial growth factor. J. Invest Dermatol 104, 7–10. 10.1111/1523-1747.ep12613450 7798644

[B5] Banno T. Gazel A. Blumenberg M. (2004). Effects of tumor necrosis factor-α (TNFα) in epidermal keratinocytes revealed using global transcriptional profiling. J. Biol. Chem. 279, 32633–32642. 10.1074/jbc.M400642200 15145954

[B6] Bashir M. M. Sharma M. R. Werth V. P. (2009a). TNF-alpha production in the skin. Arch. Dermatol Res. 301, 87–91. 10.1007/s00403-008-0893-7 18825399

[B7] Bashir M. M. Sharma M. R. Werth V. P. (2009b). UVB and proinflammatory cytokines synergistically activate TNF-alpha production in keratinocytes through enhanced gene transcription. J. Invest Dermatol 129, 994–1001. 10.1038/jid.2008.332 19005488 PMC3345265

[B8] Benais-Pont G. Punn A. Flores-Maldonado C. Eckert J. Raposo G. Fleming T. P. (2003). Identification of a tight junction-associated guanine nucleotide exchange factor that activates Rho and regulates paracellular permeability. J. Cell Biol. 160, 729–740. 10.1083/jcb.200211047 12604587 PMC2173357

[B9] Benhadou F. Glitzner E. Brisebarre A. Swedlund B. Song Y. Dubois C. (2020). Epidermal autonomous VEGFA/Flt1/Nrp1 functions mediate psoriasis-like disease. Sci. Adv. 6, eaax5849. 10.1126/sciadv.aax5849 31934626 PMC6949033

[B10] Birkenfeld J. Nalbant P. Bohl B. P. Pertz O. Hahn K. M. Bokoch G. M. (2007). GEF-H1 modulates localized RhoA activation during cytokinesis under the control of mitotic kinases. Dev. Cell 12, 699–712. 10.1016/j.devcel.2007.03.014 17488622 PMC1965589

[B11] Birukova A. A. Adyshev D. Gorshkov B. Bokoch G. M. Birukov K. G. Verin A. D. (2006). GEF-H1 is involved in agonist-induced human pulmonary endothelial barrier dysfunction. Am. J. Physiol. Lung Cell Mol. Physiol. 290, L540–L548. 10.1152/ajplung.00259.2005 16257999

[B12] Brenneisen P. Blaudschun R. Gille J. Schneider L. Hinrichs R. Wlaschek M. (2003). Essential role of an activator protein-2 (AP-2)/specificity protein 1 (Sp1) cluster in the UVB-mediated induction of the human vascular endothelial growth factor in HaCaT keratinocytes. Biochem. J. 369, 341–349. 10.1042/BJ20021032 12358602 PMC1223081

[B13] Brown L. F. Olbricht S. M. Berse B. Jackman R. W. Matsueda G. Tognazzi K. A. (1995). Overexpression of vascular permeability factor (VPF/VEGF) and its endothelial cell receptors in delayed hypersensitivity skin reactions. J. Immunol. 154, 2801–2807. 10.4049/jimmunol.154.6.2801 7876550

[B14] Brown L. F. Yeo K. T. Berse B. Yeo T. K. Senger D. R. Dvorak H. F. (1992). Expression of vascular permeability factor (vascular endothelial growth factor) by epidermal keratinocytes during wound healing. J. Exp. Med. 176, 1375–1379. 10.1084/jem.176.5.1375 1402682 PMC2119412

[B15] Calautti E. Grossi M. Mammucari C. Aoyama Y. Pirro M. Ono Y. (2002). Fyn tyrosine kinase is a downstream mediator of Rho/PRK2 function in keratinocyte cell-cell adhesion. J. Cell Biol. 156, 137–148. 10.1083/jcb.200105140 11777936 PMC2173591

[B16] Chang M. Yi L. Zhou Z. Yi X. Chen H. Liang X. (2023). GEF-H1/RhoA signaling pathway mediates pro-inflammatory effects of NF-κB on CD40L-induced pulmonary endothelial cells. Mol. Immunol. 157, 42–52. 10.1016/j.molimm.2023.03.015 36989839

[B17] Chapman S. Liu X. Meyers C. Schlegel R. McBride A. A. (2010). Human keratinocytes are efficiently immortalized by a Rho kinase inhibitor. J. Clin. Invest 120, 2619–2626. 10.1172/JCI42297 20516646 PMC2898606

[B18] Chapman S. McDermott D. H. Shen K. Jang M. K. McBride A. A. (2014). The effect of Rho kinase inhibition on long-term keratinocyte proliferation is rapid and conditional. Stem Cell Res. Ther. 5, 60. 10.1186/scrt449 24774536 PMC4055106

[B19] Cherfils J. Zeghouf M. (2013). Regulation of small GTPases by GEFs, GAPs, and GDIs. Physiol. Rev. 93, 269–309. 10.1152/physrev.00003.2012 23303910

[B20] Cullis J. Meiri D. Sandi M. J. Radulovich N. Kent O. A. Medrano M. (2014). The RhoGEF GEF-H1 is required for oncogenic RAS signaling *via* KSR-1. Cancer Cell 25, 181–195. 10.1016/j.ccr.2014.01.025 24525234

[B21] Dan Q. Shi Y. Rabani R. Venugopal S. Xiao J. Anwer S. (2019). Claudin-2 suppresses GEF-H1, RHOA, and MRTF, thereby impacting proliferation and profibrotic phenotype of tubular cells. J. Biol. Chem. 294, 15446–15465. 10.1074/jbc.RA118.006484 31481470 PMC6802501

[B22] Detmar M. Brown L. F. Claffey K. P. Yeo K. T. Kocher O. Jackman R. W. (1994). Overexpression of vascular permeability factor/vascular endothelial growth factor and its receptors in psoriasis. J. Exp. Med. 180, 1141–1146. 10.1084/jem.180.3.1141 8064230 PMC2191647

[B23] Detmar M. Brown L. F. Schön M. P. Elicker B. M. Velasco P. Richard L. (1998). Increased microvascular density and enhanced leukocyte rolling and adhesion in the skin of VEGF transgenic mice. J. Invest Dermatol 111, 1–6. 10.1046/j.1523-1747.1998.00262.x 9665379

[B24] Detmar M. Yeo K. T. Nagy J. A. Van de Water L. Brown L. F. Berse B. (1995). Keratinocyte-derived vascular permeability factor (vascular endothelial growth factor) is a potent mitogen for dermal microvascular endothelial cells. J. Invest Dermatol 105, 44–50. 10.1111/1523-1747.ep12312542 7615975

[B25] Ettehadi P. Greaves M. W. Wallach D. Aderka D. Camp R. D. (1994). Elevated tumour necrosis factor-alpha (TNF-alpha) biological activity in psoriatic skin lesions. Clin. Exp. Immunol. 96, 146–151. 10.1111/j.1365-2249.1994.tb06244.x 8149659 PMC1534536

[B26] Fine N. Dimitriou I. D. Rullo J. Sandí M. J. Petri B. Haitsma J. (2016). GEF-H1 is necessary for neutrophil shear stress-induced migration during inflammation. J. Cell Biol. 215, 107–119. 10.1083/jcb.201603109 27738004 PMC5057286

[B27] Frank S. Hübner G. Breier G. Longaker M. T. Greenhalgh D. G. Werner S. (1995). Regulation of vascular endothelial growth factor expression in cultured keratinocytes. Implications for normal and impaired wound healing. J. Biol. Chem. 270, 12607–12613. 10.1074/jbc.270.21.12607 7759509

[B28] Fujishiro S.-H. Tanimura S. Mure S. Kashimoto Y. Watanabe K. Kohno M. (2008). ERK1/2 phosphorylate GEF-H1 to enhance its guanine nucleotide exchange activity toward RhoA. Biochem. Biophys. Res. Commun. 368, 162–167. 10.1016/j.bbrc.2008.01.066 18211802

[B29] Fukazawa A. Alonso C. Kurachi K. Gupta S. Lesser C. F. McCormick B. A. (2008). GEF-H1 mediated control of NOD1 dependent NF-kappaB activation by shigella effectors. PLoS Pathog. 4, e1000228. 10.1371/journal.ppat.1000228 19043560 PMC2583055

[B30] Fukuda K. Ito Y. Amagai M. (2025). Barrier integrity and immunity: exploring the cutaneous front line in health and disease. Annu. Rev. Immunol. 43, 219–252. 10.1146/annurev-immunol-082323-030832 40279307

[B31] Gebäck T. Schulz M. M. P. Koumoutsakos P. Detmar M. (2009). TScratch: a novel and simple software tool for automated analysis of monolayer wound healing assays. Biotechniques 46, 265–274. 10.2144/000113083 19450233

[B32] Goicoechea S. M. Awadia S. Garcia-Mata R. (2014). I’m coming to GEF you: regulation of RhoGEFs during cell migration. Cell Adh Migr. 8, 535–549. 10.4161/cam.28721 25482524 PMC4594598

[B33] Grossi M. Hiou-Feige A. Tommasi Di Vignano A. Calautti E. Ostano P. Lee S. (2005). Negative control of keratinocyte differentiation by Rho/CRIK signaling coupled with up-regulation of KyoT1/2 (FHL1) expression. Proc. Natl. Acad. Sci. U. S. A. 102, 11313–11318. 10.1073/pnas.0505011102 16061799 PMC1183590

[B34] Guilluy C. Swaminathan V. Garcia-Mata R. O'Brien E. T. Superfine R. Burridge K. (2011). The rho GEFs LARG and GEF-H1 regulate the mechanical response to force on integrins. Nat. Cell Biol. 13, 722–727. 10.1038/ncb2254 21572419 PMC3107386

[B35] Guo F. Tang J. Zhou Z. Dou Y. Van Lonkhuyzen D. Gao C. (2012). GEF-H1-RhoA signaling pathway mediates LPS-Induced NF-κB transactivation and IL-8 synthesis in endothelial cells. Mol. Immunol. 50, 98–107. 10.1016/j.molimm.2011.12.009 22226472

[B36] He L. Liu R. Yue H. Ren S. Zhu G. Guo Y. (2021). Actin-granule formation is an additional step in cardiac myofibroblast differentiation. Ann. Transl. Med. 9, 165. 10.21037/atm-20-8231 33569467 PMC7867932

[B37] Hodge R. G. Ridley A. J. (2016). Regulating Rho GTPases and their regulators. Nat. Rev. Mol. Cell Biol. 17, 496–510. 10.1038/nrm.2016.67 27301673

[B38] Hu Q. Lai J. Chen H. Cai Y. Yue Z. Lin H. (2023). Reducing GEF-H1 expression inhibits renal cyst formation, inflammation, and fibrosis via RhoA signaling in nephronophthisis. Int. J. Mol. Sci. 24, 3504. 10.3390/ijms24043504 36834937 PMC9967383

[B39] Huang N. Ashrafpour H. Levine R. H. Forrest C. R. Neligan P. C. Lipa J. E. (2012). Vasorelaxation effect and mechanism of action of vascular endothelial growth Factor-165 in isolated perfused human skin flaps. J. Surg. Res. 172, 177–186. 10.1016/j.jss.2010.08.016 20934716

[B40] Huang S. Robinson J. B. Deguzman A. Bucana C. D. Fidler I. J. (2000). Blockade of nuclear factor-kappaB signaling inhibits angiogenesis and tumorigenicity of human ovarian cancer cells by suppressing expression of vascular endothelial growth factor and interleukin 8. Cancer Res. 60, 5334–5339. 11034066

[B41] Jackson B. Peyrollier K. Pedersen E. Basse A. Karlsson R. Wang Z. (2011). RhoA is dispensable for skin development, but crucial for contraction and directed migration of keratinocytes. Mol. Biol. Cell 22, 593–605. 10.1091/mbc.E09-10-0859 21209320 PMC3046057

[B42] Jiu Y. Peränen J. Schaible N. Cheng F. Eriksson J. E. Krishnan R. (2017). Vimentin intermediate filaments control actin stress fiber assembly through GEF-H1 and RhoA. J. Cell Sci. 130, 892–902. 10.1242/jcs.196881 28096473 PMC5358333

[B43] Joo E. Olson M. F. (2021). Regulation and functions of the RhoA regulatory guanine nucleotide exchange factor GEF-H1. Small GTPases 12, 358–371. 10.1080/21541248.2020.1840889 33126816 PMC8583009

[B44] Ju W. D. Schiller J. T. Kazempour M. K. Lowy D. R. (1993). TGF alpha enhances locomotion of cultured human keratinocytes. J. Invest Dermatol 100, 628–632. 8491985

[B45] Kakiashvili E. Dan Q. Vandermeer M. Zhang Y. Waheed F. Pham M. (2011). The epidermal growth factor receptor mediates tumor necrosis factor-alpha-induced activation of the ERK/GEF-H1/RhoA pathway in tubular epithelium. J. Biol. Chem. 286, 9268–9279. 10.1074/jbc.M110.179903 21212278 PMC3059019

[B46] Kaplan D. H. Igyártó B. Z. Gaspari A. A. (2012). Early immune events in the induction of allergic contact dermatitis. Nat. Rev. Immunol. 12, 114–124. 10.1038/nri3150 22240625 PMC3578582

[B47] Kiriakidis S. Andreakos E. Monaco C. Foxwell B. Feldmann M. Paleolog E. (2003). VEGF expression in human macrophages is NF-kappaB-dependent: studies using adenoviruses expressing the endogenous NF-kappaB inhibitor IkappaBalpha and a kinase-defective form of the IkappaB kinase 2. J. Cell Sci. 116, 665–674. 10.1242/jcs.00286 12538767

[B48] Köck A. Schwarz T. Kirnbauer R. Urbanski A. Perry P. Ansel J. C. (1990). Human keratinocytes are a source for tumor necrosis factor alpha: evidence for synthesis and release upon stimulation with endotoxin or ultraviolet light. J. Exp. Med. 172, 1609–1614. 10.1084/jem.172.6.1609 2258696 PMC2188768

[B49] Kolde R. (2018). Pheatmap: pretty heatmaps. R. package version. Available online at: https://github.com/raivokolde/pheatmap.

[B50] Kondo T. Ohshima T. (1996). The dynamics of inflammatory cytokines in the healing process of mouse skin wound: a preliminary study for possible wound age determination. Int. J. Leg. Med. 108, 231–236. 10.1007/BF01369816 8721421

[B51] Kristensen M. Chu C. Q. Eedy D. J. Feldmann M. Brennan F. M. Breathnach S. M. (1993). Localization of tumour necrosis factor-alpha (TNF-alpha) and its receptors in normal and psoriatic skin: epidermal cells express the 55-kD but not the 75-kD TNF receptor. Clin. Exp. Immunol. 94, 354–362. 10.1111/j.1365-2249.1993.tb03457.x 8222328 PMC1534247

[B52] Lederle W. Depner S. Schnur S. Obermueller E. Catone N. Just A. (2011). IL-6 promotes malignant growth of skin SCCs by regulating a network of autocrine and paracrine cytokines. Int. J. Cancer 128, 2803–2814. 10.1002/ijc.25621 20726000

[B53] Li L. Li Y. Zhou X. (2024). Advancements in the research of GEF-H1: Biological functions and tumor associations. Curr. Mol. Pharmacol. 17, e18761429274883. 10.2174/0118761429274883231129103220 38389417

[B54] Li M. Hener P. Zhang Z. Ganti K. P. Metzger D. Chambon P. (2009). Induction of thymic stromal lymphopoietin expression in keratinocytes is necessary for generating an atopic dermatitis upon application of the active vitamin D3 analogue MC903 on mouse skin. J. Invest Dermatol 129, 498–502. 10.1038/jid.2008.232 18650845

[B55] Liang J. Zeng X. Halifu Y. Chen W. Hu F. Wang P. (2017). Blocking RhoA/ROCK inhibits the pathogenesis of Pemphigus vulgaris by suppressing oxidative stress and apoptosis through TAK1/NOD2-mediated NF-κB pathway. Mol. Cell Biochem. 436, 151–158. 10.1007/s11010-017-3086-x 28608226

[B56] Liu Y. Zienkiewicz J. Qiao H. Gibson-Corley K. N. Boyd K. L. Veach R. A. (2022). Genomic control of inflammation in experimental atopic dermatitis. Sci. Rep. 12, 18891. 10.1038/s41598-022-23042-x 36344555 PMC9640569

[B57] Lock F. E. Hotchin N. A. (2009). Distinct roles for ROCK1 and ROCK2 in the regulation of keratinocyte differentiation. PLoS One 4, e8190. 10.1371/journal.pone.0008190 19997641 PMC2780731

[B58] Longuet-Perret I. Schmitt D. Viac J. (1998). Tumour necrosis factor-alpha is involved in the contrasting effects of ultraviolet B and ultraviolet A1 radiation on the release by normal human keratinocytes of vascular permeability factor. Br. J. Dermatol 138, 221–224. 10.1046/j.1365-2133.1998.02064.x 9602864

[B59] Ly D. L. Waheed F. Lodyga M. Speight P. Masszi A. Nakano H. (2013). Hyperosmotic stress regulates the distribution and stability of myocardin-related transcription factor, a key modulator of the cytoskeleton. Am. J. Physiol. Cell Physiol. 304, C115–C127. 10.1152/ajpcell.00290.2012 23054059 PMC3546806

[B60] Machacek M. Hodgson L. Welch C. Elliott H. Pertz O. Nalbant P. (2009). Coordination of rho GTPase activities during cell protrusion. Nature 461, 99–103. 10.1038/nature08242 19693013 PMC2885353

[B61] Man X.-Y. Yang X.-H. Cai S.-Q. Bu Z.-Y. Zheng M. (2008). Overexpression of vascular endothelial growth factor (VEGF) receptors on keratinocytes in psoriasis: regulated by calcium independent of VEGF. J. Cell Mol. Med. 12, 649–660. 10.1111/j.1582-4934.2007.00112.x 18419602 PMC3822550

[B62] Man X.-Y. Yang X.-H. Cai S.-Q. Yao Y.-G. Zheng M. (2006). Immunolocalization and expression of vascular endothelial growth factor receptors (VEGFRs) and neuropilins (NRPs) on keratinocytes in human epidermis. Mol. Med. 12, 127–136. 10.2119/2006-00024.Man 17088944 PMC1626599

[B63] Martin D. Galisteo R. Gutkind J. S. (2009). CXCL8/IL8 stimulates vascular endothelial growth factor (VEGF) expression and the autocrine activation of VEGFR2 in endothelial cells by activating NFkappaB through the CBM (Carma3/Bcl10/Malt1) complex. J. Biol. Chem. 284, 6038–6042. 10.1074/jbc.C800207200 19112107 PMC2649103

[B64] McGifford O. J. Harkin D. G. Cuttle L. (2022). Effect of Rho-Associated protein kinase inhibitors on epidermal keratinocytes: a proposed application for burn wound healing. Tissue Eng. Part B Rev. 28, 555–568. 10.1089/ten.TEB.2021.0034 34039046

[B65] McMullan R. Lax S. Robertson V. H. Radford D. J. Broad S. Watt F. M. (2003). Keratinocyte differentiation is regulated by the Rho and ROCK signaling pathway. Curr. Biol. 13, 2185–2189. 10.1016/j.cub.2003.11.050 14680635

[B66] Meiri D. Marshall C. B. Mokady D. LaRose J. Mullin M. Gingras A. C. (2014). Mechanistic insight into GPCR-mediated activation of the microtubule-associated RhoA exchange factor GEF-H1. Nat. Commun. 5, 4857. 10.1038/ncomms5857 25209408

[B67] Mills C. Hemkemeyer S. A. Alimajstorovic Z. Bowers C. Eskandarpour M. Greenwood J. (2022). Therapeutic Validation of GEF-H1 Using a *de novo* Designed Inhibitor in Models of Retinal Disease. Cells 11, 1733. 10.3390/cells11111733 35681428 PMC9179336

[B68] Mizuarai S. Yamanaka K. Kotani H. (2006). Mutant p53 induces the GEF-H1 oncogene, a guanine nucleotide exchange factor-H1 for RhoA, resulting in accelerated cell proliferation in tumor cells. Cancer Res. 66, 6319–6326. 10.1158/0008-5472.CAN-05-4629 16778209

[B69] Müller L. Keil R. Glaß M. Hatzfeld M. (2024). Plakophilin 4 controls the spatio-temporal activity of RhoA at adherens junctions to promote cortical actin ring formation and tissue tension. Cell Mol. Life Sci. 81, 291. 10.1007/s00018-024-05329-6 38970683 PMC11335210

[B70] Nalbant P. Chang Y.-C. Birkenfeld J. Chang Z.-F. Bokoch G. M. (2009). Guanine nucleotide exchange factor-H1 regulates cell migration via localized activation of RhoA at the leading edge. Mol. Biol. Cell 20, 4070–4082. 10.1091/mbc.e09-01-0041 19625450 PMC2743625

[B71] Nishida M. Miyamoto K. Abe S. Shimada M. Shimizu Y. Tsuji A. (2021). Natriuretic peptide receptor-C releases and activates guanine nucleotide-exchange factor H1 in a ligand-dependent manner. Biochem. Biophys. Res. Commun. 552, 9–16. 10.1016/j.bbrc.2021.03.028 33740666

[B72] Pagès G. Pouysségur J. (2005). Transcriptional regulation of the vascular endothelial growth factor gene--a concert of activating factors. Cardiovasc Res. 65, 564–573. 10.1016/j.cardiores.2004.09.032 15664382

[B73] Partridge M. Green M. R. Langdon J. D. Feldmann M. (1989). Production of TGF-alpha and TGF-beta by cultured keratinocytes, skin and oral squamous cell carcinomas--potential autocrine regulation of normal and malignant epithelial cell proliferation. Br. J. Cancer 60, 542–548. 10.1038/bjc.1989.310 2478181 PMC2247118

[B74] Pastar I. Stojadinovic O. Yin N. C. Ramirez H. Nusbaum A. G. Sawaya A. (2014). Epithelialization in wound healing: a comprehensive review. Adv. Wound Care (New Rochelle) 3, 445–464. 10.1089/wound.2013.0473 25032064 PMC4086220

[B75] Perrot-Applanat M. Di Benedetto M. (2012). Autocrine functions of VEGF in breast tumor cells: adhesion, survival, migration and invasion. Cell Adh Migr. 6, 547–553. 10.4161/cam.23332 23257828 PMC3547902

[B76] Pertz O. Hodgson L. Klemke R. L. Hahn K. M. (2006). Spatiotemporal dynamics of RhoA activity in migrating cells. Nature 440, 1069–1072. 10.1038/nature04665 16547516

[B77] Piipponen M. Li D. Landén N. X. (2020). The immune functions of keratinocytes in skin wound healing. Int. J. Mol. Sci. 21, 8790. 10.3390/ijms21228790 33233704 PMC7699912

[B78] Qin J. Z. Bacon P. Chaturvedi V. Nickoloff B. J. (2001). Role of NF-kappaB activity in apoptotic response of keratinocytes mediated by interferon-gamma, tumor necrosis factor-alpha, and tumor-necrosis-factor-related apoptosis-inducing ligand. J. Invest Dermatol 117, 898–907. 10.1046/j.0022-202x.2001.01477.x 11676830

[B79] Raftopoulou M. Hall A. (2004). Cell migration: rho GTPases lead the way. Dev. Biol. 265, 23–32. 10.1016/j.ydbio.2003.06.003 14697350

[B80] Rhea L. Reeb T. Adelizzi E. Garnica B. Stein A. Kollash A. (2024). ARHGAP29 promotes keratinocyte proliferation and migration *in vitro* and is dispensable for *in vivo* wound healing. Dev. Dyn. 254, 310–329. 10.1002/dvdy.759 39560169 PMC11979318

[B81] Seeger M. A. Paller A. S. (2015). The roles of growth factors in keratinocyte migration. Adv. Wound Care (New Rochelle) 4, 213–224. 10.1089/wound.2014.0540 25945284 PMC4397993

[B82] Simmons J. Gallo R. L. (2024). The central roles of keratinocytes in coordinating skin immunity. J. Investigative Dermatology 144, 2377–2398. 10.1016/j.jid.2024.06.1280 PMC11920965 39115524

[B83] Sun L. Tran N. Tang F. App H. Hirth P. McMahon G. (1998). Synthesis and biological evaluations of 3-substituted indolin-2-ones: a novel class of tyrosine kinase inhibitors that exhibit selectivity toward particular receptor tyrosine kinases. J. Med. Chem. 41, 2588–2603. 10.1021/jm980123i 9651163

[B84] Takahashi H. Shibuya M. (2005). The vascular endothelial growth factor (VEGF)/VEGF receptor system and its role under physiological and pathological conditions. Clin. Sci. (Lond) 109, 227–241. 10.1042/CS20040370 16104843

[B85] Tonami K. Kurihara Y. Arima S. Nishiyama K. Uchijima Y. Asano T. (2011). Calpain-6, a microtubule-stabilizing protein, regulates Rac1 activity and cell motility through interaction with GEF-H1. J. Cell Sci. 124, 1214–1223. 10.1242/jcs.072561 21406564

[B86] Tu C.-L. Chang W. Bikle D. D. (2011). The calcium-sensing receptor-dependent regulation of cell-cell adhesion and keratinocyte differentiation requires rho and filamin A. J. Invest Dermatol 131, 1119–1128. 10.1038/jid.2010.414 21209619 PMC3078217

[B87] Van Nieuw Amerongen G. P. Koolwijk P. Versteilen A. Van Hinsbergh V. W. M. (2003). Involvement of RhoA/Rho kinase signaling in VEGF-induced endothelial cell migration and angiogenesis *in vitro* . Arterioscler. Thromb. Vasc. Biol. 23, 211–217. 10.1161/01.atv.0000054198.68894.88 12588761

[B88] Vega F. M. Colomba A. Reymond N. Thomas M. Ridley A. J. (2012). RhoB regulates cell migration through altered focal adhesion dynamics. Open Biol. 2, 120076. 10.1098/rsob.120076 22724071 PMC3376739

[B89] Venugopal S. Dan Q. Sri Theivakadadcham V. S. Wu B. Kofler M. Layne M. D. (2024). Regulation of the RhoA exchange factor GEF-H1 by profibrotic stimuli through a positive feedback loop involving RhoA, MRTF, and Sp1. Am. J. Physiol. Cell Physiol. 327, C387–C402. 10.1152/ajpcell.00088.2024 38912734

[B90] Waheed F. Dan Q. Amoozadeh Y. Zhang Y. Tanimura S. Speight P. (2013). Central role of the exchange factor GEF-H1 in TNF-α-induced sequential activation of rac, ADAM17/TACE, and RhoA in tubular epithelial cells. Mol. Biol. Cell 24, 1068–1082. 10.1091/mbc.E12-09-0661 23389627 PMC3608494

[B91] Waheed F. Speight P. Dan Q. Garcia-Mata R. Szaszi K. (2012). Affinity precipitation of active Rho-GEFs using a GST-tagged mutant rho protein (GST-RhoA(G17A)) from epithelial cell lysates. J. Vis. Exp., 3932. 10.3791/3932 22491204 PMC3460580

[B92] Waheed F. Speight P. Kawai G. Dan Q. Kapus A. Szászi K. (2010). Extracellular signal-regulated kinase and GEF-H1 mediate depolarization-induced rho activation and paracellular permeability increase. Am. J. Physiol. Cell Physiol. 298, C1376–C1387. 10.1152/ajpcell.00408.2009 20237148 PMC3226803

[B93] Wakefield P. E. James W. D. Samlaska C. P. Meltzer M. S. (1991). Tumor necrosis factor. J. Am. Acad. Dermatol 24, 675–685. 10.1016/0190-9622(91)70102-8 1869638

[B94] Wang X. Bove A. M. Simone G. Ma B. (2020). Molecular bases of VEGFR-2-Mediated physiological function and pathological role. Front. Cell Dev. Biol. 8, 599281. 10.3389/fcell.2020.599281 33304904 PMC7701214

[B95] Wang Y. Huang L. Yang Y. Xu L. Yang J. Wu Y. (2013). Effects of autocrine vascular endothelial growth factor (VEGF) in non-small cell lung cancer cell line A549. Mol. Biol. Rep. 40, 3093–3099. 10.1007/s11033-012-2383-4 23459872

[B96] Wilgus T. A. Matthies A. M. Radek K. A. Dovi J. V. Burns A. L. Shankar R. (2005). Novel function for vascular endothelial growth factor receptor-1 on epidermal keratinocytes. Am. J. Pathol. 167, 1257–1266. 10.1016/S0002-9440(10)61213-8 16251410 PMC1603795

[B97] Wunderlich L. Paragh G. Wikonkál N. M. Bánhegyi G. Kárpáti S. Mandl J. (2008). UVB induces a biphasic response of HIF-1alpha in cultured human keratinocytes. Exp. Dermatol 17, 335–342. 10.1111/j.1600-0625.2007.00640.x 18279341

[B98] Yamahashi Y. Saito Y. Murata-Kamiya N. Hatakeyama M. (2011). Polarity-regulating kinase partitioning-defective 1b (PAR1b) phosphorylates guanine nucleotide exchange factor H1 (GEF-H1) to regulate RhoA-dependent actin cytoskeletal reorganization. J. Biol. Chem. 286, 44576–44584. 10.1074/jbc.M111.267021 22072711 PMC3248009

[B99] Yousef H. Alhajj M. Fakoya A. O. Sharma S. (2024). Anatomy, skin (integument), epidermis. StatPearls Publishing. Treasure Island (FL). 29262154

[B100] Zegers M. M. Friedl P. (2014). Rho GTPases in collective cell migration. Small GTPases 5, e28997. 10.4161/sgtp.28997 25054920 PMC4114924

[B101] Zeng H. Zhao D. Mukhopadhyay D. (2002). KDR stimulates endothelial cell migration through heterotrimeric G protein Gq/11-mediated activation of a small GTPase RhoA. J. Biol. Chem. 277, 46791–46798. 10.1074/jbc.M206133200 12244099

[B102] Zenke F. T. Krendel M. DerMardirossian C. King C. C. Bohl B. P. Bokoch G. M. (2004). p21-activated kinase 1 phosphorylates and regulates 14-3-3 binding to GEF-H1, a microtubule-localized rho exchange factor. J. Biol. Chem. 279, 18392–18400. 10.1074/jbc.M400084200 14970201

[B103] Zhao Y. Alonso C. Ballester I. Song J. H. Chang S. Y. Guleng B. (2012). Control of NOD2 and Rip2-dependent innate immune activation by GEF-H1. Inflamm. Bowel Dis. 18, 603–612. 10.1002/ibd.21851 21887730 PMC3594873

[B104] Zhu J.-W. Wu X. J. Luo D. Lu Z. F. Cai S. Q. Zheng M. (2012). Activation of VEGFR-2 signaling in response to moderate dose of ultraviolet B promotes survival of normal human keratinocytes. Int. J. Biochem. Cell Biol. 44, 246–256. 10.1016/j.biocel.2011.10.022 22062947

[B105] Zhu W. J. Li P. Wang L. Xu Y. C. (2020). Hypoxia-inducible factor-1: a potential pharmacological target to manage psoriasis. Int. Immunopharmacol. 86, 106689. 10.1016/j.intimp.2020.106689 32585606

