# Supplementary material for: Keratinocyte-derived VEGF-A is an essential pro-migratory autocrine mediator, acting through the KDR/GEF-H1/RhoA pathway
Source: Front Cell Dev Biol. 2025 Jul 17;13:1601887. doi: 10.3389/fcell.2025.1601887 (PMC12310649; doi:10.3389/fcell.2025.1601887)
Supplement: Supplementary file 1 [file Image1.pdf]

Supplemental Fig 1:

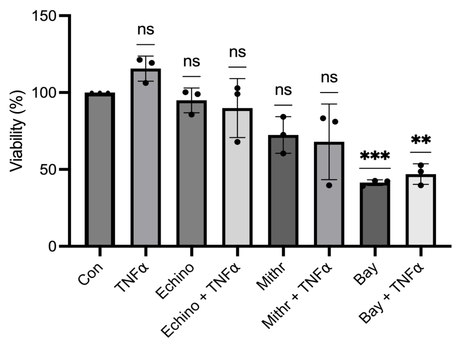

Supplemental Fig 1: Cell viability measured using MTT Assay. Inhibitors and TNFα were added for 16h (as in Fig 1G). Values are expressed as the percentage of the untreated control (100%). (n=3, one sample t-test vs. 100: ***p<0.001, **p<0.01, ns-nonsignificant)

Supplemental Fig 2:

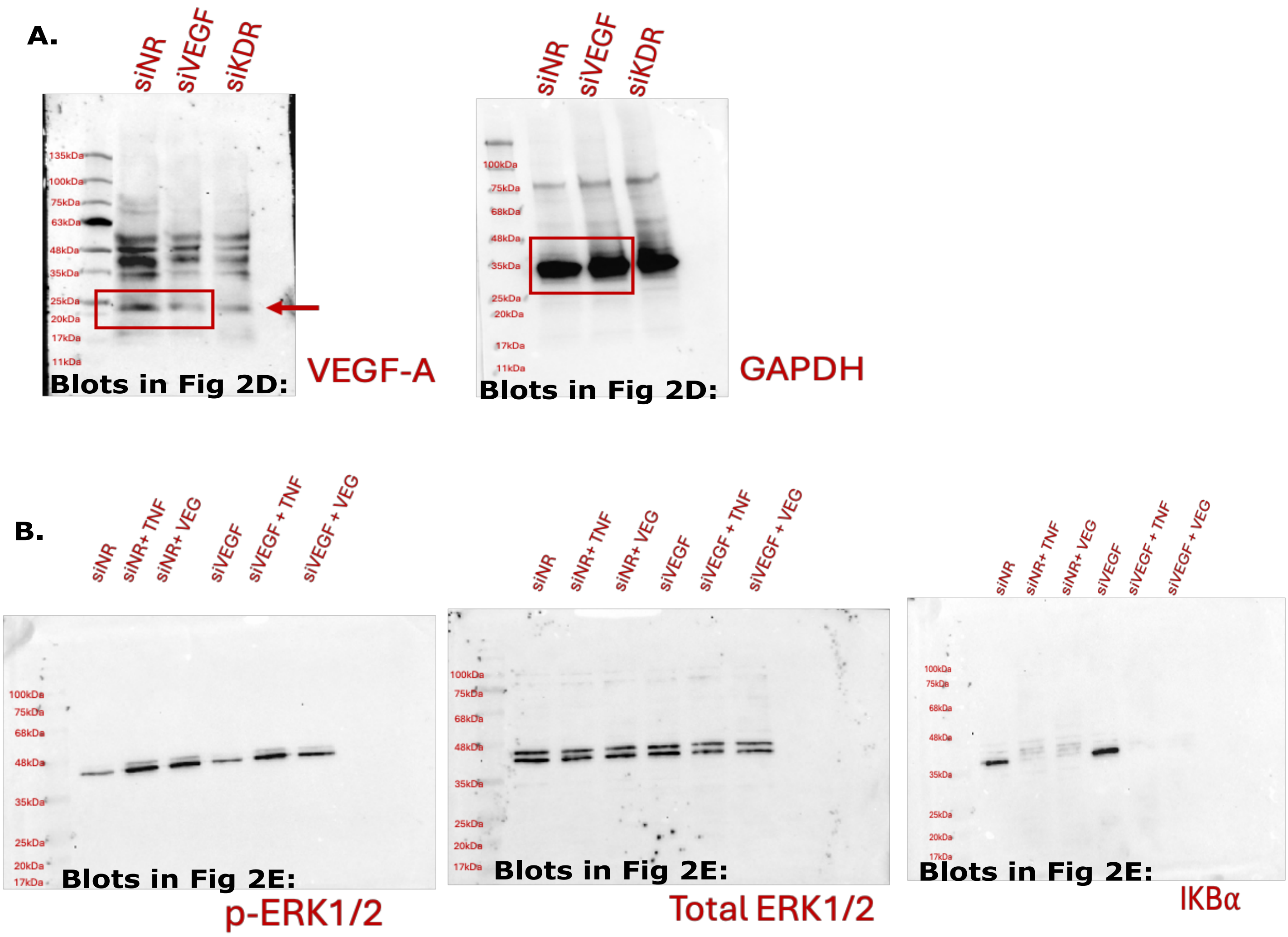

Supplemental Fig 2A-B: Original uncropped blots shown on Fig 2. The red box indicates the area shown on the figure.

Supplemental Fig 3:

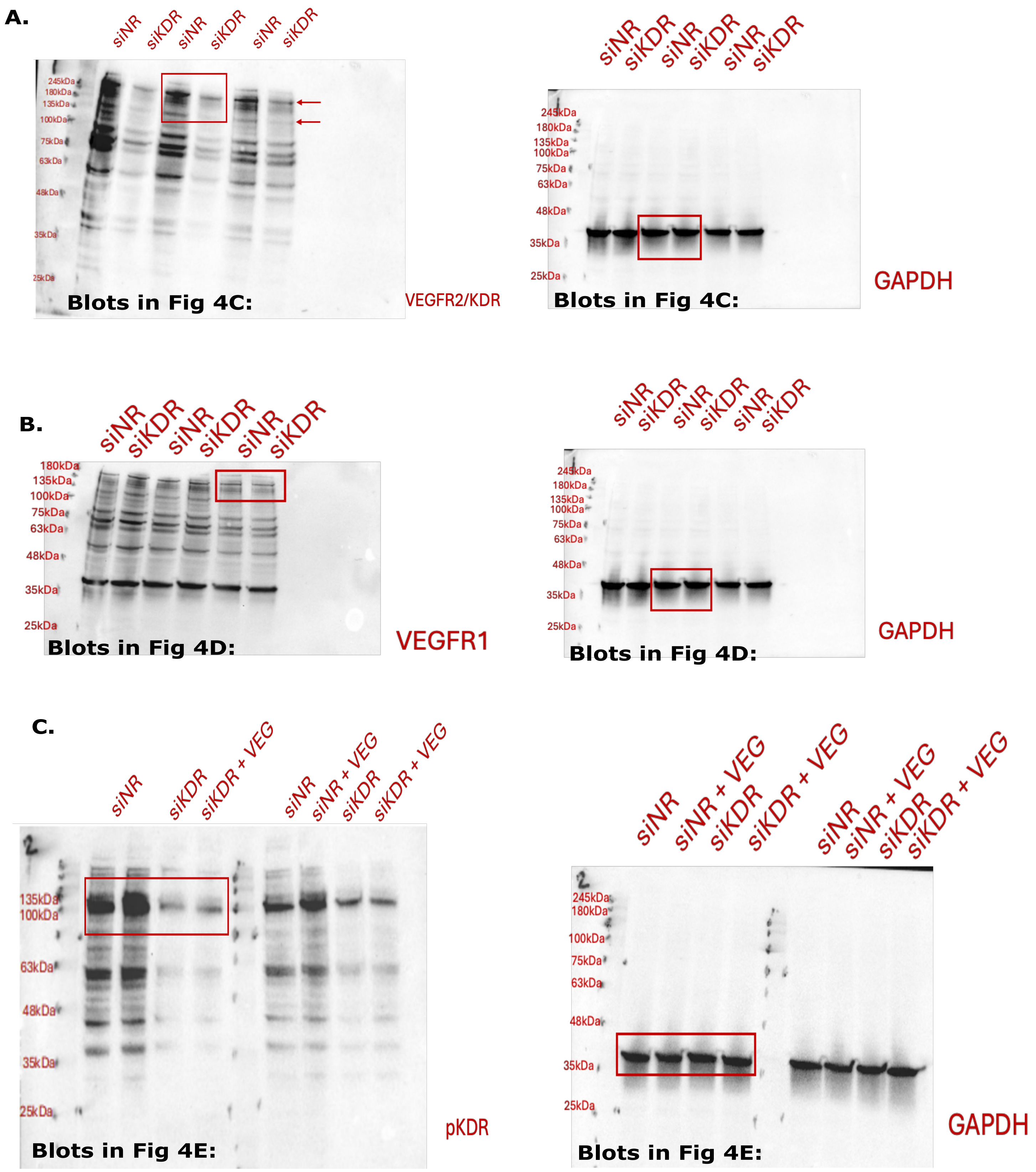

Supplemental Fig 3:

D.

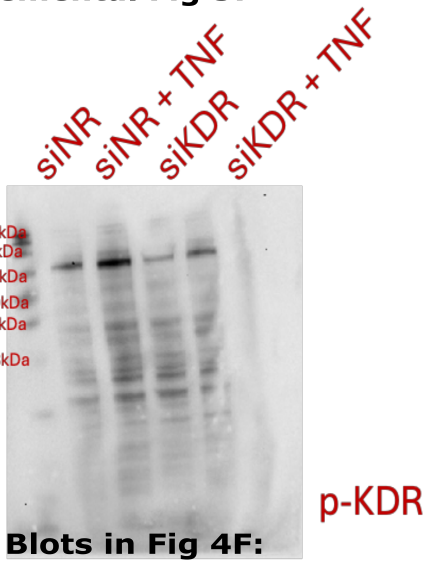

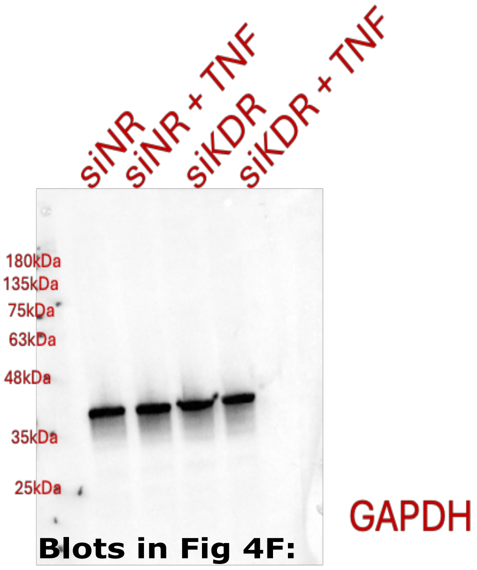

E.

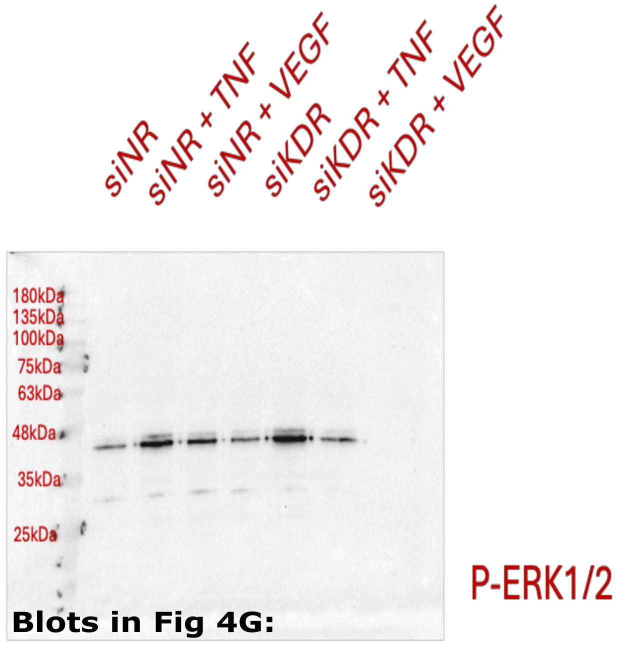

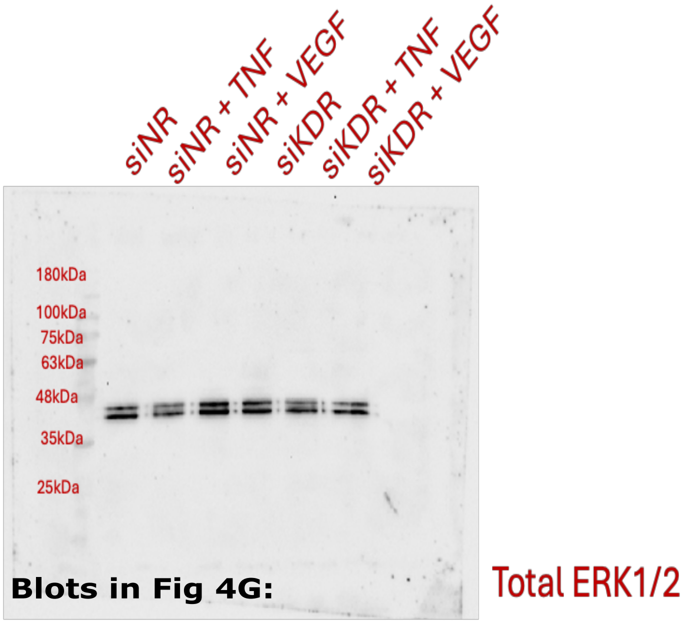

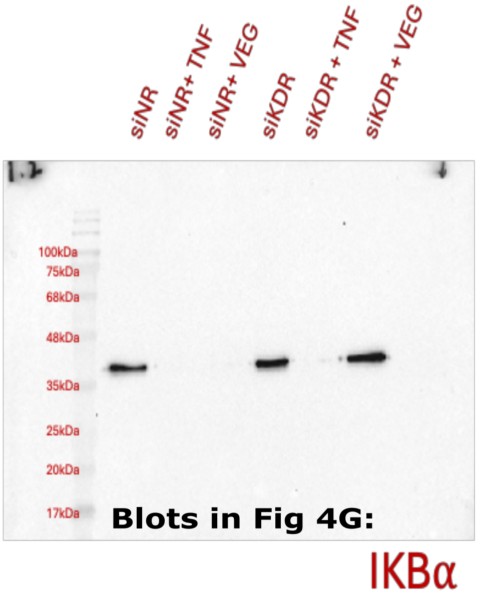

Supplemental Fig 3A-E: Original uncropped blots for Fig 4. The red boxes indicate the area shown on the figure.

Supplemental Fig 4:

A.

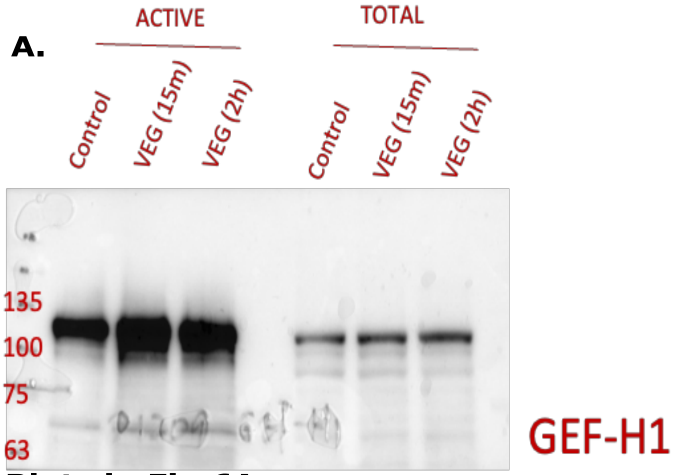

Blots in Fig 6A:

B.

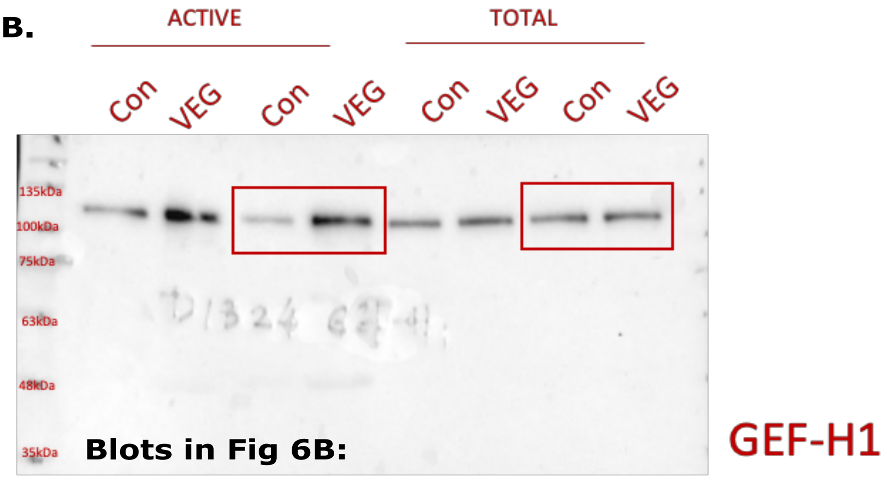

Blots in Fig 6B:

C.

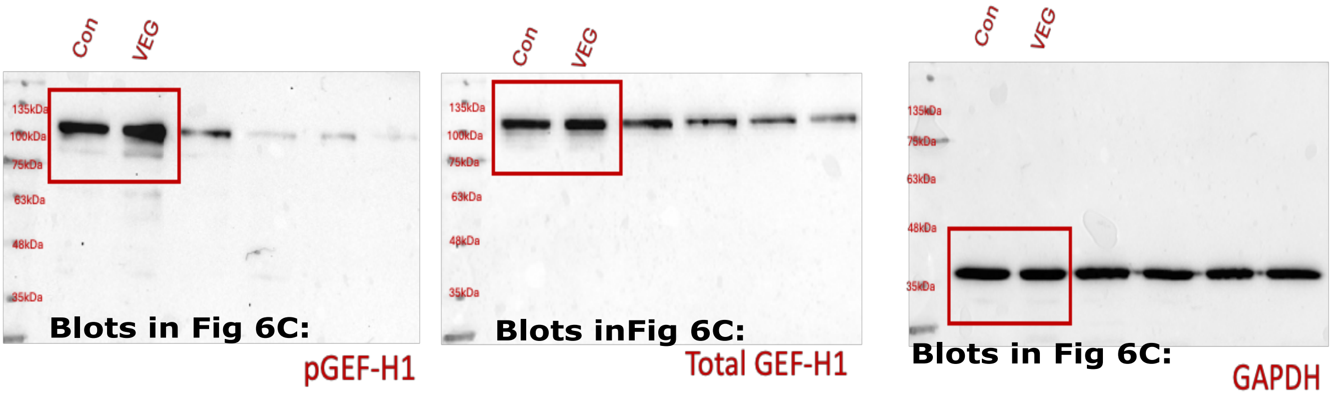

Blots in Fig 6C:

Blots inFig 6C:

Blots in Fig 6C:

Supplemental Fig 4:

D.

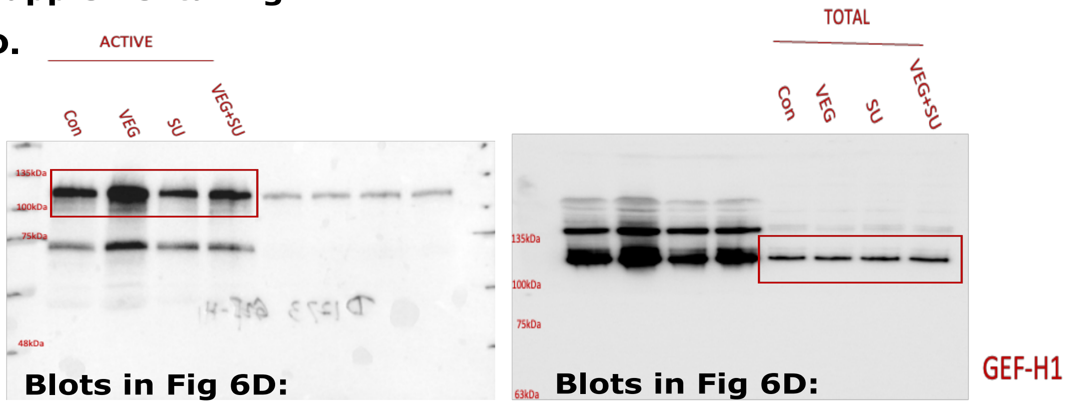

Blots in Fig 6D: **Blots in Fig 6D:**

E.

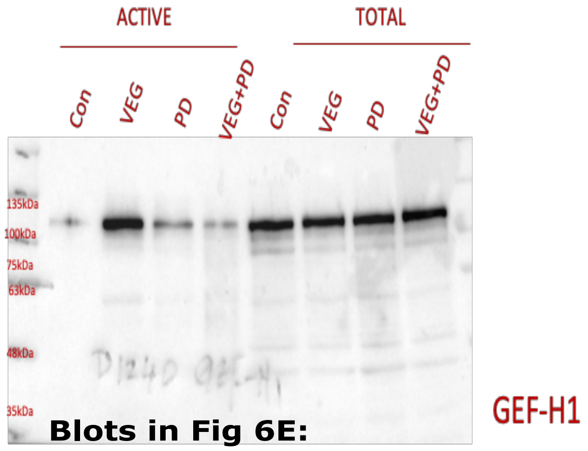

Blots in Fig 6E:

Supplemental Fig 4A-E : Original uncropped blots for Fig 6. The red boxes indicate the area shown on the figure.

Supplemental Fig 5:

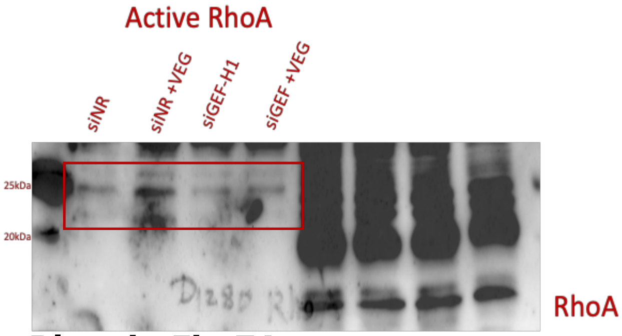

Blots in Fig 7A:

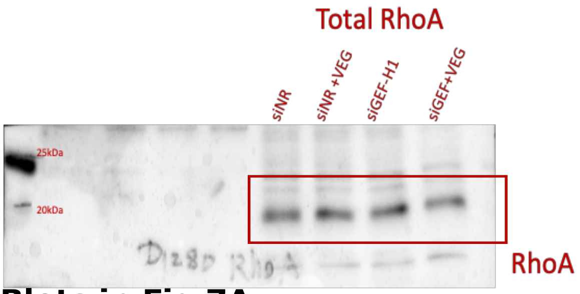

Blots in Fig 7A:

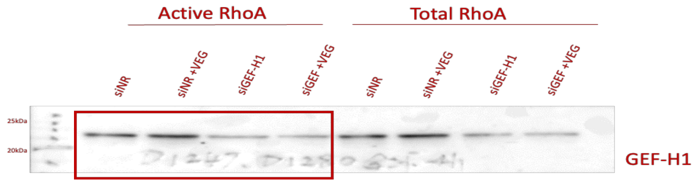

Blots in Fig 7A:

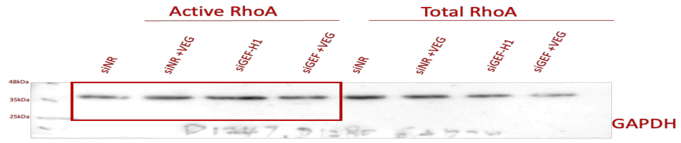

Blots in Fig 7A:

Supplemental Fig 5: Original uncropped blots for Fig 7A. The red boxes indicate the area shown on the figure.
